# Morphological, Biochemical, and Proteomic Analyses to Understand the Promotive Effects of Plant-Derived Smoke Solution on Wheat Growth under Flooding Stress

**DOI:** 10.3390/plants11111508

**Published:** 2022-06-04

**Authors:** Setsuko Komatsu, Hisateru Yamaguchi, Keisuke Hitachi, Kunihiro Tsuchida, Shafiq Ur Rehman, Toshihisa Ohno

**Affiliations:** 1 Faculty of Life and Environmental Sciences, Fukui University of Technology, Fukui 910-8505, Japan; ohno@fukui-ut.ac.jp; 2 Department of Medical Technology, Yokkaichi Nursing and Medical Care University, Yokkaichi 512-8045, Japan; h-yamaguchi@y-nm.ac.jp; 3 Institute for Comprehensive Medical Science, Fujita Health University, Toyoake 470-1192, Japan; hkeisuke@fujita-hu.ac.jp (K.H.); tsuchida@fujita-hu.ac.jp (K.T.); 4 Department of Biology, University of Haripur, Haripur 22620, Pakistan; drshafiq@yahoo.com

**Keywords:** proteomics, wheat, plant-derived smoke solution, flooding stress

## Abstract

Wheat is an important staple food crop for one-third of the global population; however, its growth is reduced by flooding. On the other hand, a plant-derived smoke solution enhances plant growth; however, its mechanism is not fully understood. To reveal the effects of the plant-derived smoke solution on wheat under flooding, morphological, biochemical, and proteomic analyses were conducted. The plant-derived smoke solution improved wheat-leaf growth, even under flooding. According to the functional categorization of proteomic results, oppositely changed proteins were correlated with photosynthesis, glycolysis, biotic stress, and amino-acid metabolism with or without the plant-derived smoke solution under flooding. Immunoblot analysis confirmed that RuBisCO activase and RuBisCO large/small subunits, which decreased under flooding, were recovered by the application of the plant-derived smoke solution. Furthermore, the contents of chlorophylls *a* and *b* significantly decreased by flooding stress; however, they were recovered by the application of the plant-derived smoke solution. In glycolysis, fructose-bisphosphate aldolase and glyceraldehyde-3-phosphate dehydrogenase decreased with the application of the plant-derived smoke solution under flooding as compared with flooding alone. Additionally, glutamine, glutamic acid, aspartic acid, and serine decreased under flooding; however, they were recovered by the plant-derived smoke solution. These results suggest that the application of the plant-derived smoke solution improves the recovery of wheat growth through the regulation of photosynthesis and glycolysis even under flooding conditions. Furthermore, the plant-derived smoke solution might promote wheat tolerance against flooding stress through the regulation of amino-acid metabolism.

## 1. Introduction

Wheat is the most important staple crop, and its availability can impact the livelihoods of almost every family globally [1]. Climate change is widely accepted and leads to many extreme climatic events related to temperature, precipitation, and other climatic conditions [2]. Climate change is a significant challenge to the agricultural production of wheat both regionally and globally [3]. Due to high rainfall, irrigation practices, and poor soil drainage, waterlogging annually affects large areas of farmlands worldwide, and these effects result in anoxic soils and severe hypoxia or anoxia within crop roots [4]. Hypoxia caused by waterlogging inhibited the growth of crop roots/stems and the yield of seeds [5]. Because waterlogging tolerance is different among wheat varieties, its tolerance mechanism during wheat growth has not been elucidated.

The plant-derived smoke solution is a material for promoting plant growth/development and affects plant species from various habitats [6]. Plant-derived smoke positively affected the post-germination growth of rice [7,8,9], maize [10], chickpea [11], soybean [12,13,14,15], and wheat [16]. Studies on the post-germination of crops treated with the plant-derived smoke solution elucidated that its treatment affected not only the seed-germination stage but also the plant growth and development stages [17]. Butanolides, including karrikins and cyanohydrin, are the active compounds in the plant-derived smoke solution [6]. The functional mechanisms of plant-derived smoke in the seed-germination stage were clarified with the discovery of karrikin [18]. A study on the molecular aspects of seed germination reported that abscisic acid, seed maturation, and dormancy-related transcripts were up-regulated by trimethyl butenolide and suppressed by karrikin 1, indicating that increased seed germination by karrikin 1 might be due to suppression of abscisic acid [6,18]. However, the role of karrikin is not elucidated for plant-growth stages. 

It was reported that the plant-derived smoke solution enhanced soybean growth under flooding [13] and after flooding [12]. Zhong et al. [13] reported that proteins related to the ubiquitin-proteasome pathway were altered and led to the sacrifice-for-survival-mechanism-driven degradation of the root tip in soybean by the plant-derived smoke solution, which enabled the accumulation of metabolites and guaranteed lateral-root development during soybean recovery from flooding. Li et al. [12] reported that the plant-derived smoke solution enhanced soybean growth during recovery from flooding through the balance of sucrose/starch metabolism and glycolysis and/or the accumulation of cell-wall-related protein. On the other hand, in the case of wheat, the plant-derived smoke treatment improved the shoot length under optimum conditions [16]; however, the mechanism, which promotes plant growth, is not fully understood. In this study, to reveal the dynamic effects of the plant-derived smoke solution on wheat under flooding, a morphological analysis was performed. Based on its result, proteomic analysis using nano-liquid chromatography (LC) and mass spectrometry (MS)/MS was conducted. Furthermore, the proteomic results were confirmed using immunoblot analysis, chlorophyll-contents assay, and amino-acid analysis.

## 2. Results

### 2.1. Morphological Changes of Wheat Treated with Plant-Derived Smoke Solution under Flooding Stress

**Figure 1:**
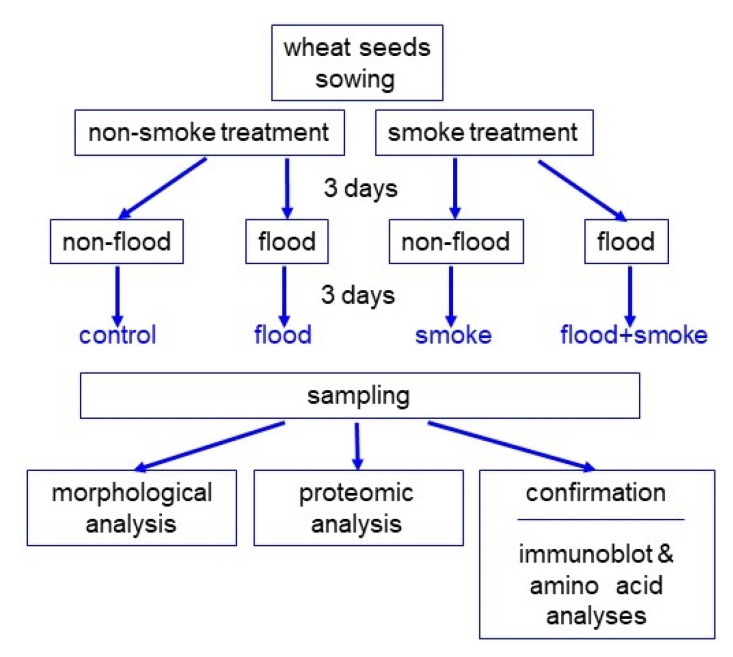
The experimental design for the investigation of the effect of the plant-derived smoke solution on wheat under flooding stress. To investigate the potential effects of the plant-derived smoke solution on wheat, seeds were sown and treated with or without 2000 ppm of the plant-derived smoke solution. After 3 days of sowing, wheat was flooded for 3 days. Wheat seedlings were analyzed with morphological and proteomic methods, and confirmation. For confirmation experiments, immunoblot and amino-acid analyses were used. All experiments were performed with three independent biological replicates.

**Figure 2:**
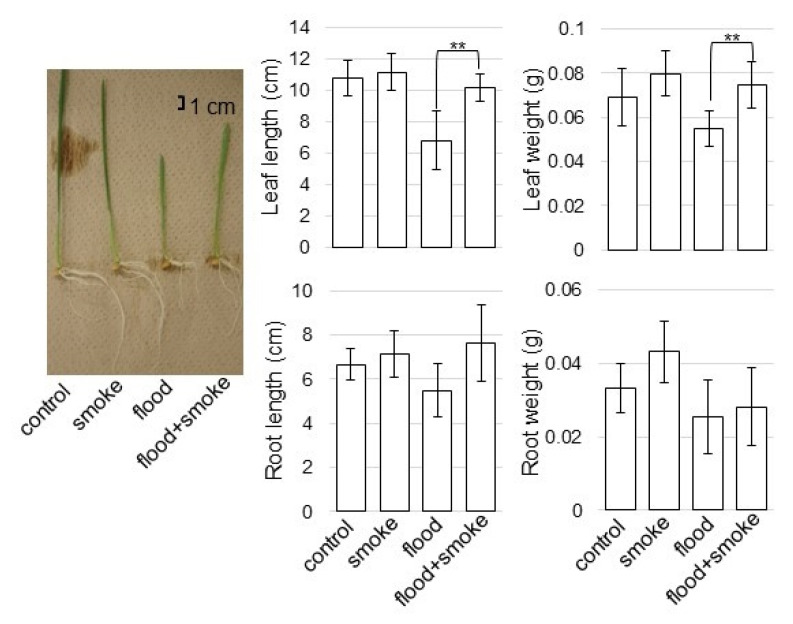
The morphological effects of the plant-derived smoke solution on wheat under flooding stress. Wheat seeds were sown and treated with or without 2000 ppm of the plant-derived smoke solution. Three-day-old wheats were treated with or without flooding for three days. As morphological parameters, leaf length, leaf-fresh weight, main-root length, and total-root fresh weight were analyzed 6 days after sowing. The bar in the left panel indicates 1 cm in the picture. The data are presented as mean ± SD from three independent biological replicates. Asterisks indicate significant changes between wheats treated with the plant-derived smoke solution under flooding and with only flooding according to the Student’s *t*-test (**: *p* < 0.01).

To investigate the effect of the plant-derived smoke solution on wheat under flooding stress, morphological analysis was performed. Wheat seeds were treated with 2000 ppm of the plant-derived smoke solution, and the 3-day-old plant was flooded for 3 days (Figure 1). As morphological parameters, leaf length, leaf-fresh weight, main-root length, and total-root fresh weight were measured (Figure 2). All parameters decreased under flooding; however, leaf length and leaf-fresh weight increased with the application of the plant-derived smoke solution, even if it was under flooding (Figure 2). Based on the morphological results, wheat leaves were used for proteomic analysis.

### 2.2. Protein Identification and Functional Categorization in Wheat Treated with Plant-Derived Smoke Solution under Flooding Stress

To investigate the cellular mechanism in wheat growth by the application of the plant-derived smoke solution under flooding stress, a gel-free/label-free proteomics was conducted (Table S1). Three kinds of treatments, which were control, flood, and flood + smoke, were performed. Proteins extracted from wheat leaves after treatment were enriched, reduced, alkylated, and digested. After analysis by LC combined MS/MS, the relative abundance of proteins from without (Table S2) or with (Table S3) the plant-derived smoke solution under flooding stress was compared to that from the control. 

**Figure 3:**
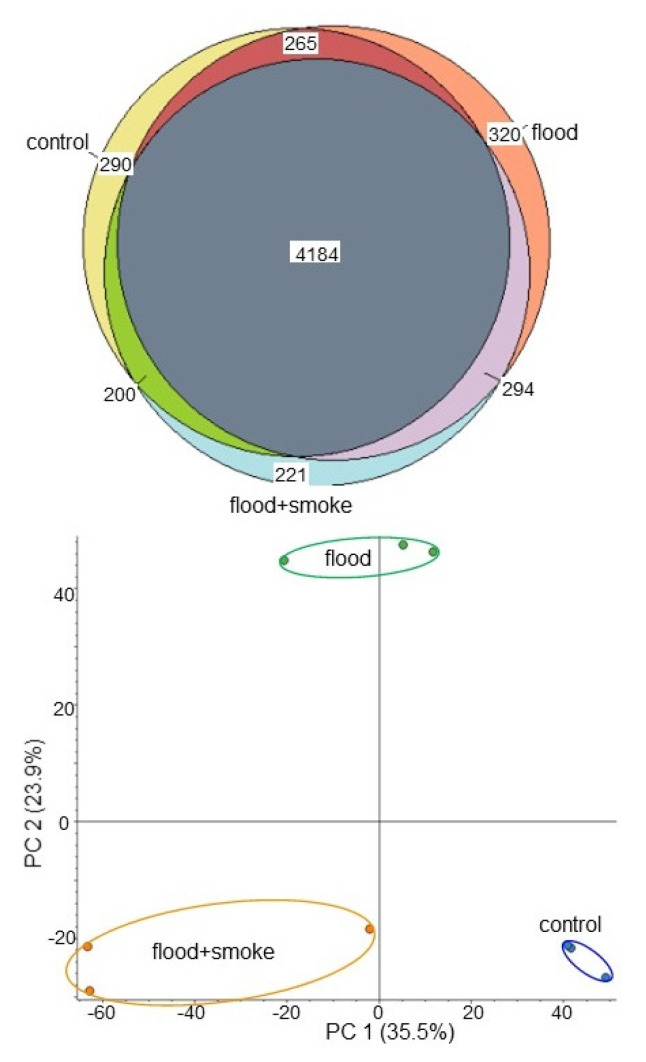
A Venn diagram of the proteomic results and an overview of the proteomic data of wheat based on PCA. Wheat seeds were sown and treated with or without the plant-derived smoke solution. Three-day-old wheats were exposed with or without flooding for 3 days. Wheat leaves were collected for protein extraction. Proteomic analysis was performed with 3 independent biological replicates for each treatment. The number in the Venn diagram shows the number of proteins identified by proteomic analysis. PCA was performed with Proteome Discoverer 2.2 using proteins from 9 kinds of samples.

Totally, 5774 proteins were identified by LC-MS/MS analysis (Figure 3). The proteomic results of all 9 samples from different 3 groups were compared by principal component analysis (PCA), which showed the different accumulation patterns of proteins from three different kinds of treatment (Figure 3). This result indicated that flooding stress largely affected the wheat proteins; however, this effect was recovered at the protein level by the application of the plant-derived smoke solution, even if it was under flooding (Figure 3). 

**Figure 4:**
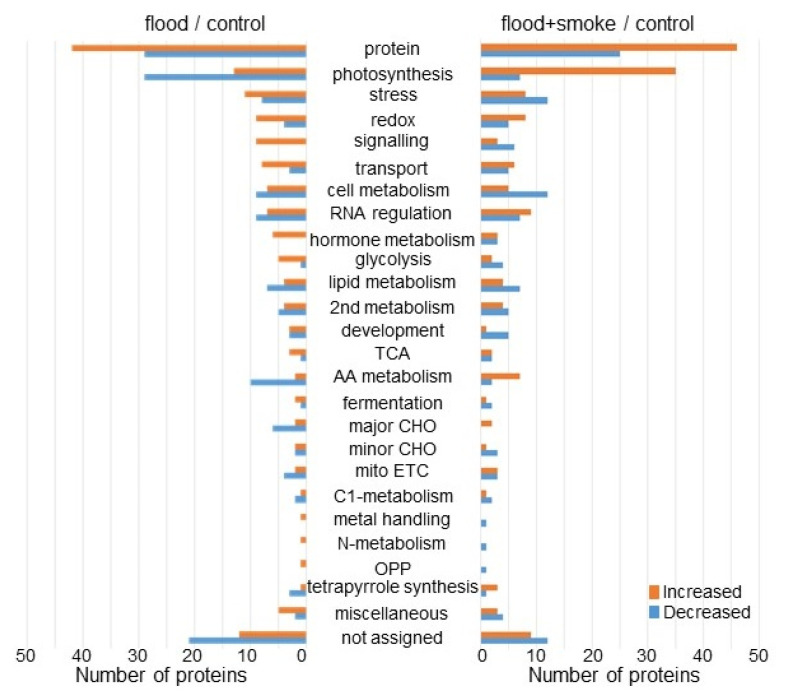
The functional categories of proteins with differential abundance in wheat treated with the plant-derived smoke solution under flooding stress. Wheat seeds were sown and treated with or without the plant-derived smoke solution. Three-day-old wheats were exposed with or without flooding. After proteomic analysis, the functional categories of the significantly changed proteins (*p* < 0.05) from wheat treated with and without the plant-derived smoke solution under flooding were determined using MapMan bin codes (Tables S2 and S3). Red and blue columns show the number of increased and decreased proteins, respectively. Abbreviations: AA, amino acids; mitoETC, mitochondrial electron transport chain; OPP, oxidative pentose phosphate; and TCA, tricarboxylic acid cycle; “not assigned” indicates proteins without ontology or characterized functions.

The abundance of 314 proteins differentially changed with the *p*-value < 0.05 and fold change >1.5 and <2/3 in wheat leaves under flooding compared to the control condition (Table S2). Among the 314 proteins, 173 and 141 proteins increased and decreased, respectively, under flooding stress compared to the control condition (Table S2 and Figure 4 left). On the other hand, the abundance of another 349 proteins also differentially changed with the *p*-value < 0.05 and fold change >1.5 and <2/3 in wheat leaves applied the plant-derived smoke solution under flooding compared to the control condition (Table S3). Among these 349 proteins, 169 and 180 proteins increased and decreased, respectively, with the application of the plant-derived smoke solution under flooding compared to the control condition (Table S3 and Figure 4 right). The functional category of identified proteins was obtained using MapMan bin codes (Figure 4). The abundance of proteins related to photosynthesis, glycolysis, and amino-acid metabolism was oppositely changed between the flood/control and flood + smoke/control. To confirm the results obtained from the proteomic analysis, oppositely changed functional categories, which are photosynthesis, glycolysis, and amino-acid metabolism, were further analyzed using immunoblot and amino-acid analyses.

### 2.3. Immunoblot Analysis of Proteins Related to Photosynthesis in Wheat Treated with Plant-Derived Smoke Solution under Flooding Stress

**Figure 5:**
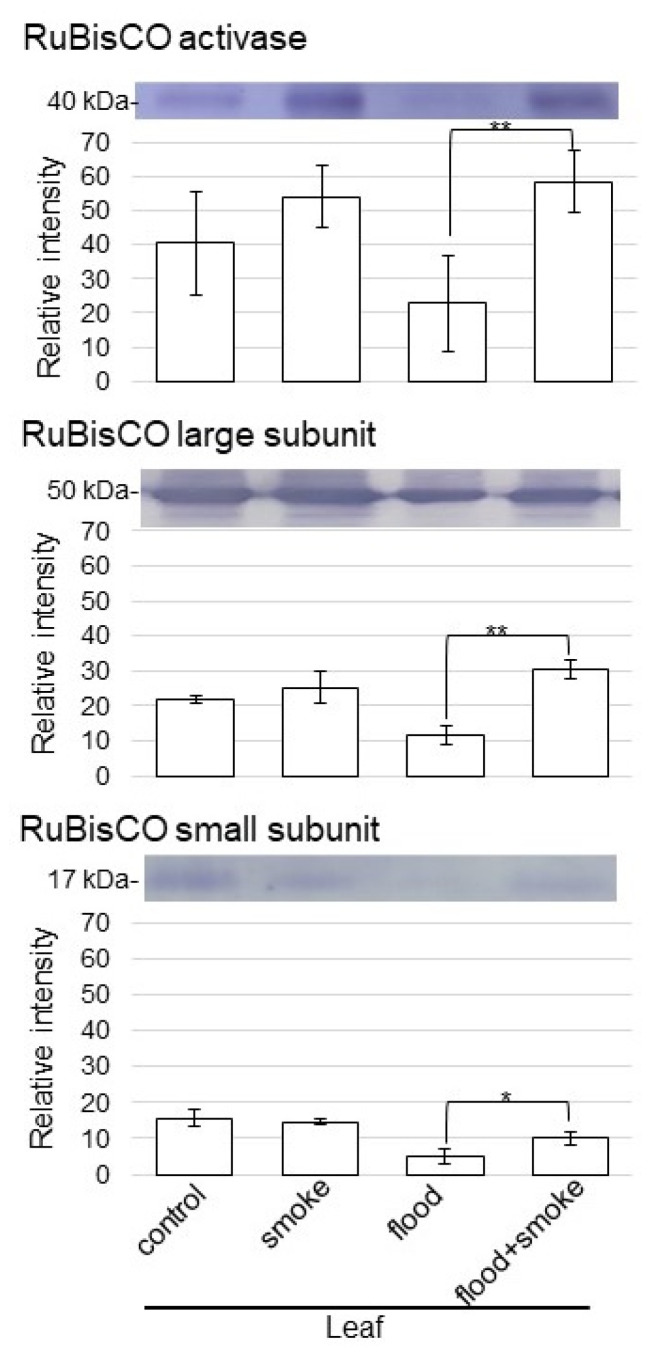
Immunoblot analysis of the proteins involved in photosynthesis in wheat treated with the plant-derived smoke solution under flooding stress. Proteins extracted from leaves of wheat seedlings were separated on SDS-polyacrylamide gel by electrophoresis and transferred onto membranes. The membranes were cross-reacted with anti-RuBisCO activase, the RuBisCO large subunit, and the RuBisCO small subunit antibodies. A staining pattern with Coomassie-brilliant blue was used as a loading control (Figure S1). The integrated densities of the bands were calculated using ImageJ software. The data are presented as mean ± SD from 3 independent biological replicates (Figure S2). Asterisks indicate significant changes in the relative intensity of signal band in the plant-derived smoke solution under flooding compared to only flooding according to the Student’s *t*-test (**, *p* < 0.01; *, *p* < 0.05).

As proteins related to photosynthesis were altered in wheat with the application of the plant-derived smoke solution under flooding stress, the abundance of the ribulose-bisphosphate carboxylase/oxygenase (RuBisCO) activase, the RuBisCO large subunit, and the RuBisCO small subunit was selectively analyzed using immunoblot analysis (Figure 5). Proteins extracted from wheat leaves were separated on the SDS-polyacrylamide gel by electrophoresis and transferred onto membranes. The membranes were cross-reacted with anti-RuBisCO activase, the RuBisCO large subunit, and the RuBisCO small subunit antibodies. A staining pattern with Coomassie-brilliant blue was used as a loading control (Figure S1). The integrated densities of bands were calculated using ImageJ software with triplicated immunoblot results (Figure S2). The abundance of the RuBisCO activase, the RuBisCO large subunit, and the RuBisCO small subunit decreased under flooding stress; however, they recovered with the application of the plant-derived smoke solution under flooding (Figure 5). These results indicated that photosynthesis was improved by the plant-derived smoke solution, even if it was under flooding conditions.

### 2.4. Chlorophyll Contents in Wheat Treated with Plant-Derived Smoke Solution under Flooding Stress

**Figure 6:**
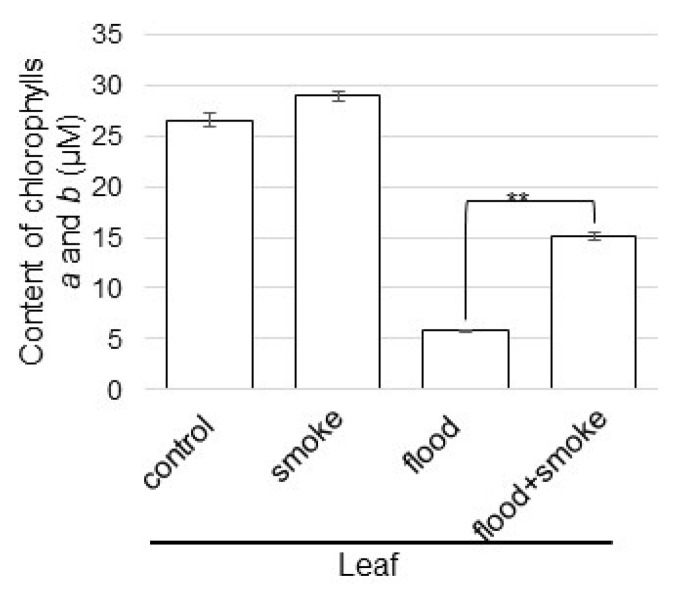
The contents of chlorophylls *a* and *b* in wheat treated with the plant-derived smoke solution under flooding stress. Chlorophylls *a* and *b* extracted from the leaves of wheat seedlings were measured. Asterisks indicate significant changes in the relative intensity of the signal band in the plant-derived smoke solution under flooding compared to only flooding according to the Student’s *t*-test (**, *p* < 0.01).

Using proteomic analysis, because proteins related to photosynthesis were altered in wheat with the application of the plant-derived smoke solution under flooding stress, the chlorophyll contents were analyzed as photosynthesis parameters (Figure 6). The contents of chlorophylls *a* and *b* significantly decreased by flooding stress; however, they were recovered by the application of the plant-derived smoke solution (Figure 6). These results indicated that photosynthesis was improved by the plant-derived smoke solution, even if it was under flooding conditions.

### 2.5. Immunoblot Analysis of Proteins Related to Glycolysis in Wheat Treated with Plant-Derived Smoke Solution under Flooding Stress

**Figure 7:**
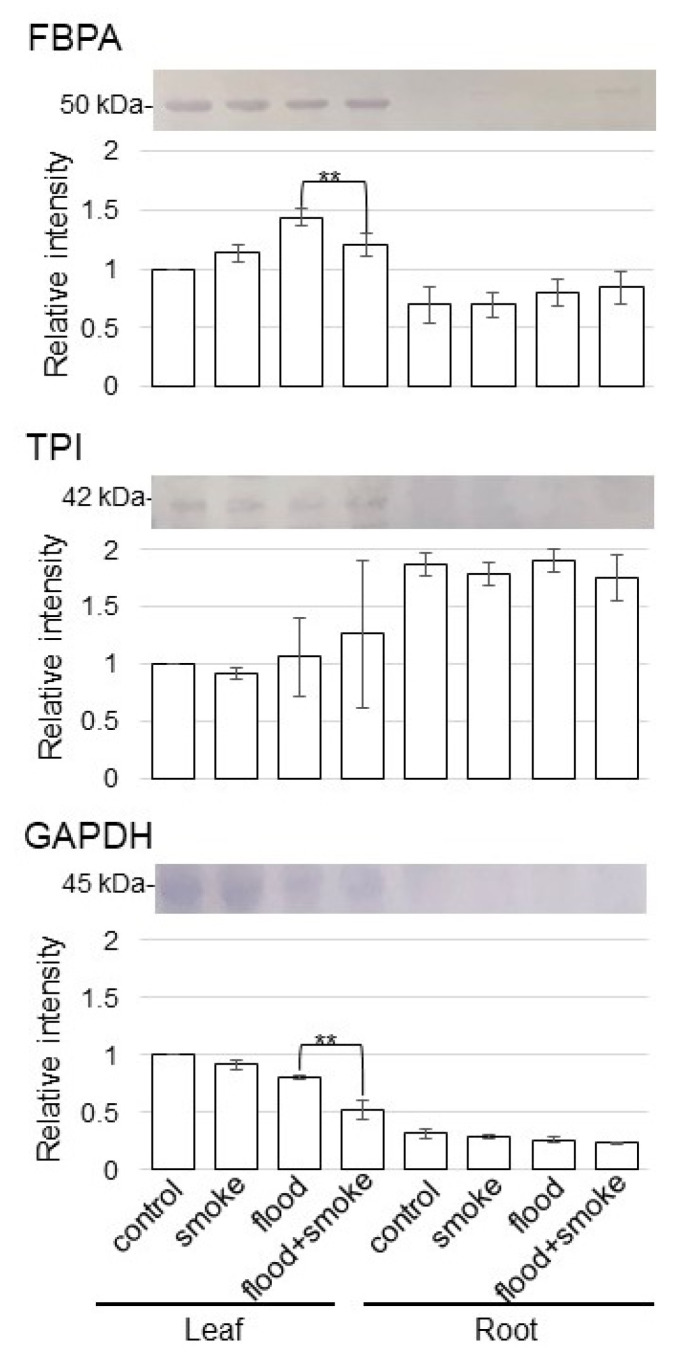
Immunoblot analysis of the proteins involved in glycolysis in wheat treated with the plant-derived smoke solution under flooding stress. Proteins extracted from the leaves and roots of wheat seedlings were separated on SDS-polyacrylamide gel by electrophoresis and transferred onto membranes. The membranes were cross-reacted with anti-FBPA, TPI, and GAPDH antibodies. A staining pattern with Coomassie-brilliant blue was used as a loading control (Figure S1). The integrated densities of bands were calculated using ImageJ software. The data are presented as mean ± SD from 3 independent biological replicates (Figures S3–S5). Asterisks indicate significant changes in the relative intensity of the signal band in the plant-derived smoke solution under flooding compared to only flooding according to the Student’s *t*-test (**: *p* < 0.01).

As proteins related to glycolysis were altered in wheat with the application of the plant-derived smoke solution under flooding stress, the abundance of fructose-bisphosphate aldolase (FBPA), triose-phosphate isomerase (TPI), and glyceraldehyde-3-phosphate dehydrogenase (GAPDH) was selectively analyzed using the immunoblot analysis (Figure 7). Proteins extracted from the leaves and roots of wheat were separated on SDS-polyacrylamide gel by electrophoresis and transferred onto membranes. The membranes were cross-reacted with anti-FBPA, TPI, and GAPDH antibodies. A staining pattern with Coomassie-brilliant blue was used as a loading control (Figure S1). The integrated densities of bands were calculated using ImageJ software with triplicated immunoblot results (Figures S3–S5). The abundance of FBPA increased by flooding stress; however, it was recovered in wheat leaves with the application of the plant-derived smoke solution (Figure 7). The abundance of GAPDH decreased by flooding stress, and it was further decreased in wheat leaves with the application of the plant-derived smoke solution (Figure 7). On the other hand, TPI did not change with the application of the plant-derived smoke solution (Figure 7). These results indicated that the balance of glycolysis-related proteins, which were FBPA and GAPDH, was affected by the plant-derived smoke solution.

### 2.6. Immunoblot Analysis of Proteins Related to Biotic Stress in Wheat Treated with Plant-Derived Smoke Solution under Flooding Stress

**Figure 8:**
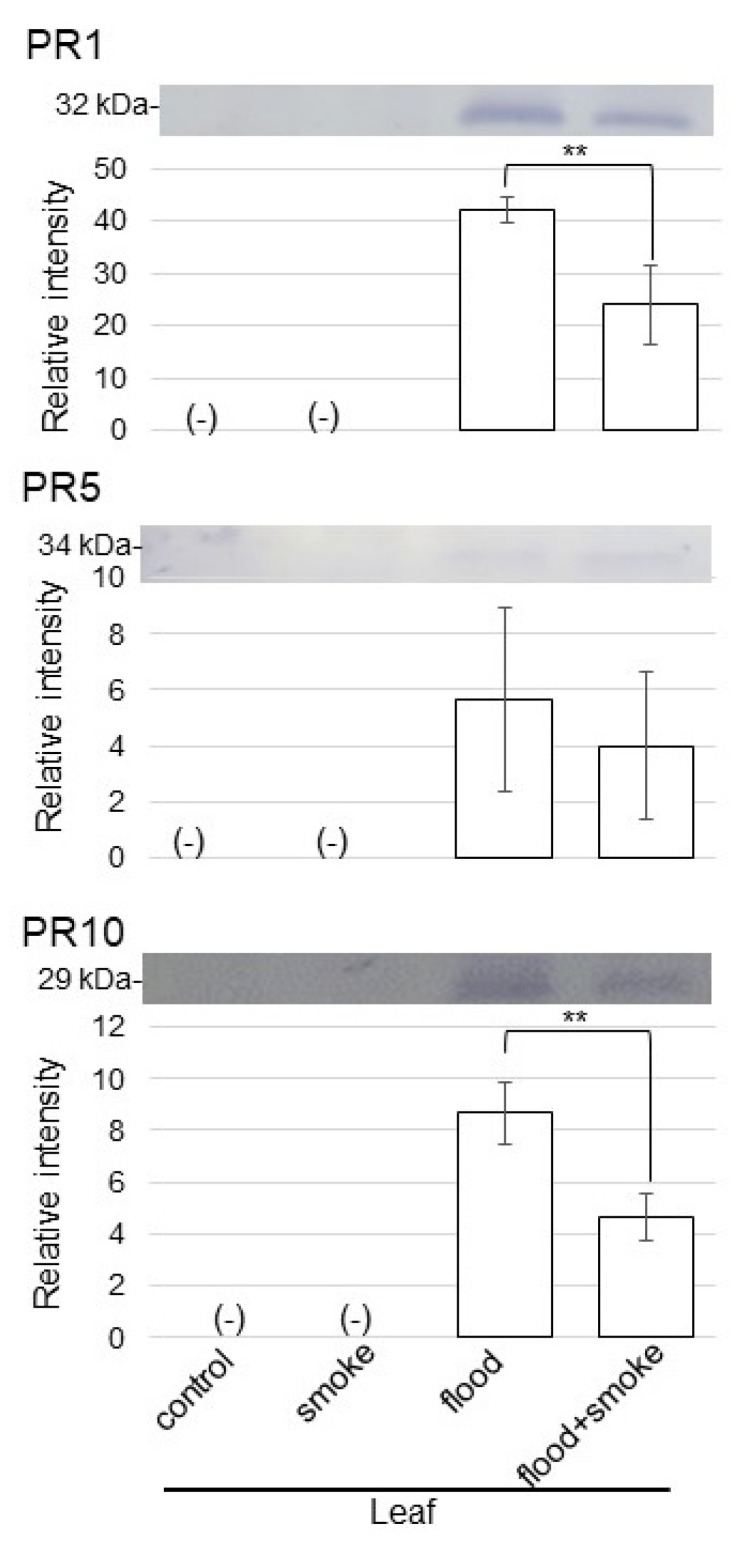
Immunoblot analysis of the proteins involved in biotic stress in wheat treated with the plant-derived smoke solution under flooding stress. Proteins extracted from the leaves of wheat seedlings were separated on SDS-polyacrylamide gel by electrophoresis and transferred onto membranes. The membranes were cross-reacted with anti-PR1, PR5, and PR10 antibodies. A staining pattern with Coomassie-brilliant blue was used as a loading control (Figure S1). The integrated densities of the bands were calculated using ImageJ software. The data are presented as mean ± SD from 3 independent biological replicates (Figures S3–S5). Asterisks indicate significant changes in the relative intensity of signal band in the plant-derived smoke solution under flooding compared to only flooding according to the Student’s *t*-test (**: *p* < 0.01).

Using proteomic analysis, the abundance of pathogen-related protein (PR)-1 and PR 10 increased under flooding stress (Table S2) and decreased with the application of the plant-derived smoke solution (Table S3) in wheat leaves. On the other hand, thaumatin, named PR5, mildly increased under flooding stress and significantly increased by the application of the plant-derived smoke solution under flooding stress (Tables S2 and S3). As proteins related to biotic stress were altered in wheat with the application of the plant-derived smoke solution under flooding stress, the abundance of PR1, PR5, and PR10 was selectively analyzed using immunoblot analysis (Figure 8). Proteins extracted from wheat leaves were separated on SDS-polyacrylamide gel by electrophoresis and transferred onto membranes. The membranes were cross-reacted with anti-PR1, PR5, and PR10 antibodies. A staining pattern with Coomassie-brilliant blue was used as a loading control (Figure S1). The integrated densities of the bands were calculated using ImageJ software with triplicated immunoblot results. The abundance of the PR1, PR5, and PR10 increased under flooding stress; however, it was recovered with the application of the plant-derived smoke solution under flooding (Figure 8). These results indicated that biotic stress was suppressed by the plant-derived smoke solution, even if it was under flooding conditions. 

### 2.7. Amino-Acid Analysis in Wheat Treated with Plant-Derived Smoke Solution under Flooding Stress

**Figure 9:**
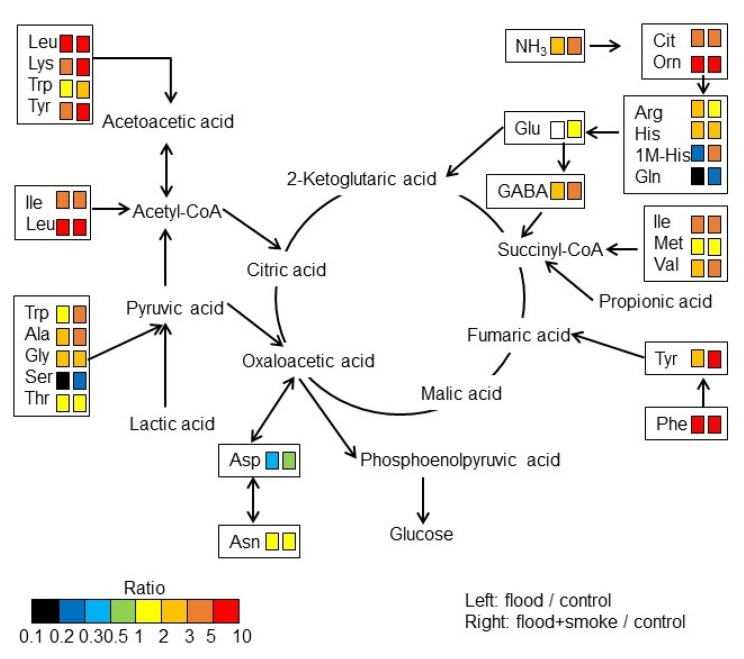
A mapping of altered amino acids to amino-acid metabolism in wheat treated with the plant-derived smoke solution under flooding stress. Totally, 32 amino acids identified using an automatic amino-acid analyzer were mapped onto pathways according to the KEGG database. Amino-acids analysis was performed with 3 independent biological replicates for each treatment (Table S4). The different colors indicate the different ratio ranges of the quantities of metabolites, which are calculated using the contents of wheat treated with or without the plant-derived smoke solution under flooding by those from untreated wheat. Each set of 2 boxes shows that the left is “flood/control” and the right is “flood + smoke/control”. Abbreviations: GABA, gamma-aminobutyric acid.

As proteins related to amino-acid metabolism were altered in wheat with the treatment of the plant-derived smoke solution under flooding stress, the abundance of amino acids was analyzed using the automatic amino-acid analyzer. In total, 32 amino acids were identified in wheat (Table S4) and mapped on amino-acid metabolism using the KEGG database (Figure 9). In altered amino acids, the abundance of glutamine (Gln), glutamic acid (Glu), aspartic acid (Asp), and serine (Ser) decreased under flooding; however, it was recovered by the application of the plant-derived smoke solution. The abundance of ornithine and phenylalanine (Phe) significantly increased under flooding; however, it was recovered by the application of the plant-derived smoke solution. The abundance of alanine (Ala), citrulline, valine (Val), and gamma-aminobutyric acid (GABA) increased under flooding; it further increased with the application of the plant-derived smoke solution (Figure 9). These results indicated that amino-acid metabolism was significantly affected by the plant-derived smoke solution under flooding.

## 3. Discussion

### 3.1. Plant-Derived Smoke Solution Improves Flooding Tolerance of Wheat

The use of organic fertilizers and plant-derived herbicides for seed or plant treatment is the sensible example of efforts in the direction of sustainable agricultural practices. This result shows an increasing demand for such naturally derived agro-chemicals for sustainable farming systems. It was reported that root/shoot length, fresh weight, and dry weight, as well as leaf area, were improved by the plant-derived smoke solution in wheat under optimum conditions [16]. However, in the present study, the leaf length/weight and root length/weight of wheat were not improved by the plant-derived smoke solution under optimum conditions (Figure 2). It might be affected by the differences of the materials of smoke solution between *Cymbopogon jwarncusa* used in this study and plants used in the previous study, which were rice, *Cynodon dactylon*, *Pongamia glabra*, *Populus deltoides*, and *Morus alba* [16]. In the case of soybean, the plant-derived smoke solution prepared from *Cymbopogon jwarncusa* increased the length of the root, including hypocotyl under optimum conditions, although its weight did not change [13]. Because the effect of the plant-derived smoke solution is not the same among plant species [6], the effect on plant growth might be different between soybean and wheat.

Karrikin, which is one component of the plant-derived smoke solution [18], regulated tolerance to abiotic stresses such as drought [19] and cold [20] in *Arabidopsis thaliana* as well as salt [21] and cadmium [22] in oil plant. Furthermore, it was reported that the plant-derived smoke solution enhanced soybean growth under flooding [13] and after flooding [12]. In this study, the length and weight of wheat leaves increased by the application of plant-derived smoke under flooding stress (Figure 2). This result with previous findings suggests that the plant-derived smoke solution has a positive effect against abiotic stress, including flooding stress.

### 3.2. Photosynthesis Activity Increases in Wheat by Plant-Derived Smoke Solution under Flooding

The combined solution of *Bacillus safensis* and plant-derived smoke prepared from *Cymbopogon jwarancusa* primed seeds increased the germination percentage, seedling growth, ion contents, and photosynthetic pigments, such as chlorophyll *a* and chlorophyll *b* [23]. Most proteins involved by karrikin are related to photosynthesis, carbohydrate metabolism, redox homeostasis, transcription control, proteosynthesis, and protein metabolism in *Arabidopsis thaliana* [24]. Li et al. [25] reported that cytokinin and brassinosteroid metabolism was specifically regulated by the D14, strigolactone receptor *dwarf14*, pathway, whereas the photosynthesis and metabolism of glucosinolates and trehalose were potentially regulated by both D14 and KAI2, karrikin receptor *karrikin insensitive 2*, pathways in plant response to water scarcity. In this study, the abundance of RuBisCO activase and RuBisCO large/small subunits decreased by flooding stress; however, they were recovered in wheat with the application of the plant-derived smoke solution (Figure 5). Furthermore, the contents of chlorophyll *a* and chlorophyll *b* were also recovered in wheat with the application of the plant-derived smoke solution (Figure 6). These results with the previous report suggest that the plant-derived smoke solution improved wheat-leaf growth through photosynthesis activation, even if it was under flooding.

### 3.3. Glycolysis Is Suppressed in Wheat by Plant-Derived Smoke Solution under Flooding

Glycolysis and gluconeogenesis were activated to generate energy for soybean-plant survival under anaerobic conditions [26]. In the case of chickpea, FBPA increased, while phosphoglycerate mutase decreased in glycolysis by the plant-derived smoke solution under optimum condition [11]. On the other hand, sucrose/starch metabolism and glycolysis were suppressed in soybean treated with the plant-derived smoke solution under flooding compared to flooded soybean [12]. In this study, FBPA and GAPDH decreased in wheat leaves by the application of plant-derived smoke under flooding (Figure 7). FBPA and GAPDH are located at the key intersections between glycolysis and the pentose-phosphate pathway, which are required for both pathways and are essential for the synthesis of glucose [27]. Additionally, both FBPA and GAPDH exert so-called “moonlighting” functions in yeast and other organisms, which are biological activities in addition to their catalytic role in glycolysis and gluconeogenesis [28]. Plants overcome their oxygen limitations and adapt by reducing alcohol fermentation, which is toxic to plants because of the production of ethanol, and by enhancing glycolysis. However, plant-derived smoke suppresses the glycolysis pathway in wheat, which might mildly generate the energy for surviving long-term under flooding stress. 

### 3.4. PR Proteins Are Accumulated in Wheat under Flooding and Suppressed by Plant-Derived Smoke Solution under Flooding

PR proteins are an integral part of the defense mechanisms of plants against various types of abiotic and biotic stresses [29]. Plants evolved different kinds of defense mechanisms, including physical and chemical defenses, to protect themselves from pathogens, which terminate pathogen infection and disease development [30]. Flooding stress limits the flow of light to plants, induces hypoxia in plants, and increases their vulnerability to pathogen attacks [31,32]. Interacted proteins with SUB1A, which is the master regulator of submergence tolerance, improved the crosstalk between submergence stress and pathogen defense and the modulation of elongation, respectively [33]. In this study, PR proteins increased under flooding stress and decreased with the application of the plant-derived smoke solution under flooding stress (Figure 8). The present result with previous reports indicates that flooding stress increases the vulnerability to pathogen attack and that the pathogen-defense system is important to recovering from flooding stress.

### 3.5. Amino Acids Are Accumulated by Flooding and Suppressed by the Application of Plant-Derived Smoke Solution

The ability of plant-derived smoke to act as a plant growth inducer in many species has led to widespread interest in plant biology. Karrikin was identified as the main component of plant-derived smoke formed from the reaction of sugars with amino acids [34]. Flooding resulted in a marked decrease of asparagine (Asn), which is the most abundant amino acid, and a concomitant accumulation of GABA [35]. In the present study, the abundance of Asn and GABA decreased and increased, respectively (Figure 8). The submergence inhibited photosystem II photochemistry and stimulated the breakdown of protein and the accumulation of several amino acids in rice. The accumulation of five amino acids such as arginine (Arg), Phe, proline (Pro), threonine (Thr), and Val was highly elevated in response to submergence in a submergence-sensitive line [31]. When the plant was exposed to desubmergence, the amount of each amino acid gradually declined, which reached the level of non-stress plants more rapidly in a submergence-tolerant line [36]. In this study, Arg, Phe, Thr, and Val also accelerated in wheat by flooding; on the other hand, additional the plant-derived smoke solution suppressed the accumulation of Arg (Figure 9). The abundance of Ala and tyrosine (Tyr) were elevated by submergence, but the accumulation of these amino acids was more abundant in submergence-sensitive line under the stress [36]. In this study, the abundance of Ala and Tyr increased under flooding and further increased by the application of plant-derived smoke (Figure 9). This result with the previous finding suggests that plant-derived smoke contributes to the metabolism of amino acids for the survival of wheat from flooding. 

Asp participates in glycolysis, the conjugation of indole-3-acetic acid/ethylene, and the cross-talk between salicylic acid/ jasmonic acid, indicating that the primary features for plant growth and immune control are N recycling, translocation, and signaling [37]. The physiological impacts of Asp in plants are uncovering the conspicuous roles of Asp in regulating the plant adaptation and tolerance to abiotic and biotic stress cues [38]. The responsive behavior of the primary metabolism in association with energy processing, including glycolysis and the pentose-phosphate pathway, ATP, the tricarboxylic acid (TCA) cycle, and the biosynthesis of amino acids, requires for energy production (Lys and Met) and photorespiration (Glu, Arg, Ser, and Gly) responding various stress cues [39]. In this study, Asp significantly decreased under flooding stress and recovered with the application of the plant-derived smoke solution (Figure 9), suggesting that plant-derived smoke can rescue wheat from flooding stress. 

Glutamate plays a central role in amino acid metabolism, in particular, in aminotransferase reactions leading to the formation of many other proteinogenic and nonproteinogenic amino acids. In stress conditions, glutamate can be either metabolized to GABA by glutamate decarboxylase, which initiates a GABA shunt bypassing several reactions of the TCA cycle, or converted to 2-oxoglutarate by glutamate dehydrogenase [40]. GABA plays a dual role in regulating the C:N balance and nitrogen metabolism, as well as being involved in many physiological processes, such as carbon flux in TCA cycle and the antioxidant effect. Furthermore, GABA acts as an important signal that triggers a series of downstream responses, such as cold or salt stress tolerance; regulates cytoplasmic pH; and controls programmed cell death [41,42]. Concurrently, verifying the altered accumulation of amino acids such as Glu, Asp, Asn, Pro, and GABA prompted the potential to be a defense indicator, aiding in uncovering the synergistically fine-tuned Asp pathway upon flooding stress.

## 4. Materials and Methods

### 4.1. Plant Material and Treatment

The plant-derived smoke solution was prepared from semi-dried *Cymbopogon jwarncusa* (Kohat University of Science and Technology, Kohat, Pakistan) [13], which was modified from previous methods [43]. The seeds of wheat (*Triticum aestivum* L. cultivar Nourin 61; Asahi Noen Seed, Inasawa, Japan) were sterilized with 2% sodium hypochlorite solution, rinsed with water, and sown with or without 2000 ppm plant-derived smoke in 400 mL of silica sand in a seedling case. Plants were grown in a growth chamber with white fluorescent light (16 h light of 200 µmol m^−2^ s^−1^ and 8 h dark photoperiod) with 60% humidity at 25 °C. Three-day-old plants were flooded for three days. Leaf length, leaf-fresh weight, main-root length, and total-root fresh weight were measured 6 days after sowing. Three independent experiments were performed as biological replicates for all experiments. In each experiment, 20 seeds were sown for each replication of each treatment. For the morphological experiment, 10 seedlings were collected for each replication of each treatment. For other biological experiments, 5–10 seedlings were collected for each replication of each treatment. The sowing of seeds was carried out on different days for making biological replicates. 

### 4.2. Protein Extraction

A portion (300 mg) of leaves of wheat was excised into small pieces and put into a filter cartridge (Cosmo Bio, Carlsbad, CA, USA). It was ground with a plastic rod 120 times in 75 μL of lysis buffer, which contained 7 M urea, 2 M thiourea, 5% CHAPS, and 2 mM tributylphosphine. The suspension was incubated for 2 min at 25 °C and centrifuged twice with 15,000× *g* at 4 °C for 5 min. The detergents from the supernatant were removed using the Pierce Detergent Removal Spin Column (Pierce Biotechnology, Rockford, IL, USA). The protein concentration was determined with the Bradford method [44] with the bovine serum albumin as the standard. Quantified proteins were used for proteomic and immunoblot analyses.

### 4.3. Protein Enrichment, Reduction, Alkylation, and Digestion

Extracted proteins (100 µg) were adjusted to a final volume of 100 µL. To each sample was added 400 µL of methanol, and it was mixed before the addition of 100 µL of chloroform and 300 µL of water. After centrifugation at 20,000× *g* for 10 min, the upper phase was discarded and 300 µL of methanol was added to the lower phase. After centrifugation at 20,000× *g* for 10 min, the pellet was resuspended in 50 mM NH_4_HCO_3_, reduced with 50 mM dithiothreitol for 30 min at 56 °C, and alkylated with 50 mM iodoacetamide for 30 min at 37 °C in the dark. Alkylated proteins were digested with trypsin and lysyl endopeptidase (Wako, Osaka, Japan) at a 1:100 enzyme/protein ratio for 16 h at 37 °C. Peptides were desalted with MonoSpin C18 Column (GL Sciences, Tokyo, Japan) and acidified with 1% trifluoroacetic acid [45].

### 4.4. Protein Identification Using LC-MS/MS

Peptides were analyzed by LC (EASY-nLC 1000; Thermo Fisher Scientific, San Jose, CA, USA) combined with MS/MS (Orbitrap Fusion ETD MS; Thermo Fisher Scientific, San Jose, CA, USA) as described in the previous study [46] (Table S1). The peptides were loaded onto the LC system equipped with a trap column (Acclaim PepMap 100 C18 LC column, 3 µm, 75 µm ID × 20 mm; Thermo Fisher Scientific, San Jose, CA, USA) equilibrated with 0.1% formic acid and eluted with a linear acetonitrile gradient (0–35%) in 0.1% formic acid at a flow rate of 300 nL/min. The eluted peptides were loaded and separated on the column (EASY-Spray C18 LC column, 3 µm, 75 µm ID x 150 mm; Thermo Fisher Scientific, San Jose, CA, USA) with a spray voltage of 2 kV (Ion Transfer Tube temperature: 275 °C). The peptide ions were detected using MS in the data-dependent acquisition mode with the installed Xcalibur software (version 4.0; Thermo Fisher Scientific, San Jose, CA, USA). Full-scan mass spectra were acquired in the MS over 375–1500 m/z with a resolution of 120,000. The most intense precursor ions were selected for collision-induced fragmentation in the linear ion trap at a normalized collision energy of 35%. Dynamic exclusion was employed within 60 sec to prevent the repetitive selection of peptides.

### 4.5. Analysis of MS/MS Data

The MS/MS searches were carried out using MASCOT (version 2.6.1, Matrix Science, London, UK) and SEQUEST HT search algorithms against the UniProtKB *Triticum aestivum* protein database (25 October 2017) using Proteome Discoverer 2.2 (version 2.2.0.388; Thermo Scientific, San Jose, CA, USA). The condition of analysis is described in the previous study [13] (Table S1). The workflow for both algorithms included spectrum files RC, spectrum selector, MASCOT, SEQUEST HT search nodes, percolator, ptmRS, and minor feature detector nodes. The oxidation of methionine was set as a variable modification, and the carbamidomethylation of cysteine was set as a fixed modification. MS and MS/MS mass tolerances were set to 10 ppm and 0.6 Da, respectively. Trypsin was specified as protease, and a maximum of one missed cleavage was allowed. Target-decoy database searches were used for the calculation of the false discovery rate, which was set at 1% for peptide identification.

### 4.6. Differential Analysis of Proteins Using MS Data

Label-free quantification was performed with Proteome Discoverer 2.2 using precursor ions quantifiler nodes. Principal component analysis (PCA) was also performed with Proteome Discoverer 2.2. For the differential analysis of the relative abundance of peptides and proteins between samples, the freely software Perseus (version 1.6.2.3, Max Planck Institute of biochemistry, Martinsried, Germany) was used. The condition of analysis is described in the previous study [47]. Proteins and peptides abundances were transferred into the log2 scale. Three biological replicates of each sample were grouped, and a minimum of three valid values were required in at least one group. The normalization of the abundances was performed to subtract the median of each sample. Missing values were imputed based on a normal distribution (width = 0.3, down-shift = 1.8). The significance was assessed using *t*-test analysis.

### 4.7. Immunoblot Analysis

Proteins extracted from leaves and roots were added in an SDS-sample buffer consisting of 60 mM Tris-HCl (pH 6.8), 2% SDS, 10% glycerol, and 50 mM dithiothreitol as the final concentration [48]. Proteins (10 µg) were separated by electrophoresis on a 10% SDS-polyacrylamide gel and transferred onto a polyvinylidene difluoride membrane using a semidry transfer blotter (Nippon Eido, Tokyo, Japan). The blotted membrane was blocked for 5 min in Bullet Blocking One reagent (Nacalai Tesque, Kyoto, Japan). After blocking, the membrane was cross-reacted with a 1:1000 dilution of the primary antibodies for 30 min. As the primary antibodies, the followings were used: anti-ribulose bisphosphate carboxylase/oxygenase (RuBisCO) activase [49]; RuBisCO large subunit [50]; RuBisCO small subunit [50]; fructose-bisphosphate aldolase (FBPA) [51]; triose-phosphate isomerase (TPI) [52]; glyceraldehyde-3-phosphate dehydrogenase (GAPDH) [52]; and the pathogen-related protein (PR) 1 [53], PR5 [53], and PR10 [53] antibodies. As the secondary antibody, anti-rabbit IgG conjugated with horseradish peroxidase (Bio-Rad, Hercules, CA, USA) was used for 30 min incubation. The signals were detected using the TMB Membrane Peroxidase Substrate kit (Seracare, Milford, MA, USA). Coomassie brilliant blue staining was used as a loading control. The integrated densities of bands were calculated using Image J software (version 1.53e with Java 1.8.0_172; National Institutes of Health, Bethesda, MD, USA). 

### 4.8. Contents of Chlorophylls a and b

A portion (500 mg) of leaves of wheat was submerged in 1 mL of in N,N-dimethylformamide for 16 h at 4 °C. The absorbance of chlorophylls *a* and *b* released in the solvent was measured at 663.8 nm and 646.8 nm. Using absorbance, the contents of chlorophylls *a* and *b* were calculated as follows: chlorophylls *a* and *b* (µM) = 19.4 × A_646.8_ + 8.05 ×A_663.8_ [54]. 

### 4.9. Amino-Acid Analysis

A portion (500 mg) of leaves of wheat was ground in phosphate-buffered saline, including 140 mM NaCl, 2.7 mM KCl, and 10 mM PO_4_^3-,^ using a mortar and pestle. The suspension was centrifuged at 20,000× *g* for 20 min at 4 °C, and the supernatant was re-centrifuged with the same condition. The final supernatant was mixed with the same amount of 3% sulfosalicylic acid and centrifuged 20,000× *g* for 20 min at 4 °C to remove precipitated proteins. After filtration, the amino-acid concentrations in supernatants obtained were analyzed with ninhydrin regent using a fully automatic amino-acid analyzer (JLC-500/V; JEOL, Tokyo, Japan). 

### 4.10. Statistical Analysis, Gene Annotation, and Metabolite Mapping

The statistical significance of the data was analyzed by the Student’s *t*-test. A *p*-value of less than 0.05 was considered statistically significant. The gene functional annotations and protein categorization were analyzed using MapMan bin codes [55]. Amino acids were mapped using the KEGG (Kyoto Encyclopedia of Genes and Genomes) database (https://www.genome.jp/kegg/mapper.html; 4 June 2022).

## 5. Conclusions

**Figure 10:**
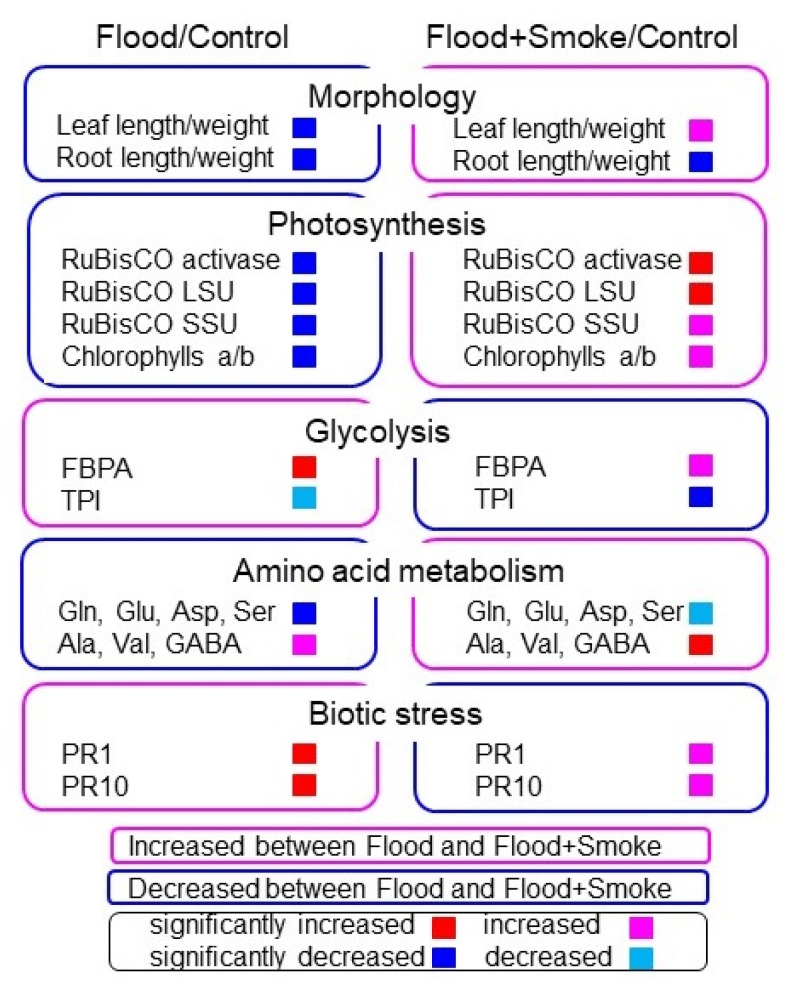
The overall responses of the main proteins in the functional categories in wheat leaf to the plant-derived smoke solution under flooding stress.

Wheat is a highly adaptable food crop that is grown extensively around the world; however, its growth is reduced by flooding. Currently, it was reported that the plant-derived smoke solution enhances soybean growth under flooding [12,13]; however, its growth-promoting mechanism is not clearly understood. The present study identified that the plant-derived smoke solution improved the wheat growth, even if it was under flooding. To reveal the role of the plant-derived smoke solution in wheat under flooding, a gel-free/label-free proteomic analysis was conducted and the results were further confirmed using biochemical techniques (Figure 10). The main findings are as follows: (i) according to a functional categorization, oppositely changed proteins were correlated with photosynthesis, glycolysis, biotic stress, and amino-acid metabolism between with and without the plant-derived smoke solution under flooding.; (ii) immunoblot analysis confirmed that RuBisCO activase and RuBisCO large/small subunits decreased in leaves under flooding and recovered by the application of the plant-derived smoke solution; (iii) in glycolysis-related proteins, FBPA and GAPDH decreased by the application of the plant-derived smoke solution under flooding compared with flooding alone.; (iv) PR1 and PR10 increased under flooding stress and recovered by the application of the plant-derived smoke solution., and (v) amino-acid analysis confirmed that Gln, Glu, Asp, and Ser decreased by flooding and recovered by the plant-derived smoke solution. These results suggest that the application of plant-derived smoke to wheat improves the recovery of plant growth through the regulation of photosynthesis, and glycolysis. Furthermore, plant-derived smoke might promote wheat tolerance against flooding and biotic stresses through the regulation of amino-acid metabolism.

## Data Availability

For MS data, RAW data, peak lists, and result files have been deposited in the ProteomeXchange Consortium [56] via the jPOST [57] partner repository under data-set identifiers PXD017690.

## Supplementary Materials

The following supporting information can be downloaded at: https://www.mdpi.com/article/10.3390/plants11111508/s1, Table S1. The experimental procedure of gel-free/label-free proteomics used in this research [12,46,47]. Table S2. A list of changed proteins in wheat leaves treated with flooding compared with control. Table S3. A list of changed proteins in wheat leaves treated with flooding and the plant-derived smoke solution compared with control. Table S4. A list of amino acids in wheat leaves treated with or without the plant-derived smoke solution under flooding compared with control. Figure S1. The Coomassie brilliant blue staining patterns of proteins used for immuno-blot analysis. Figure S2. Blots of the entire membrane with anti-RuBisCO activase, the RuBisCO large subunit, and the RuBisCO small subunit antibodies, which are used in Figure 5. Figure S3. Blots of the entire membrane with anti-FBPA antibody, which are used in Figure 7. Figure S4. Blots of the entire membrane with anti-TPI antibody, which are used in Figure 7. Figure S5. Blots of the entire membrane with anti-GAPDH antibody, which are used in Figure 7.
