# Supplementary material for: Morphological, Biochemical, and Proteomic Analyses to Understand the Promotive Effects of Plant-Derived Smoke Solution on Wheat Growth under Flooding Stress
Source: Plants (Basel). 2022 Jun 4;11(11):1508. doi: 10.3390/plants11111508 (PMC9183026; doi:10.3390/plants11111508)
Supplement: Supplementary file 1 [file plants-11-01508-s001.zip › plants-1698507-supplementary Figures.pdf]

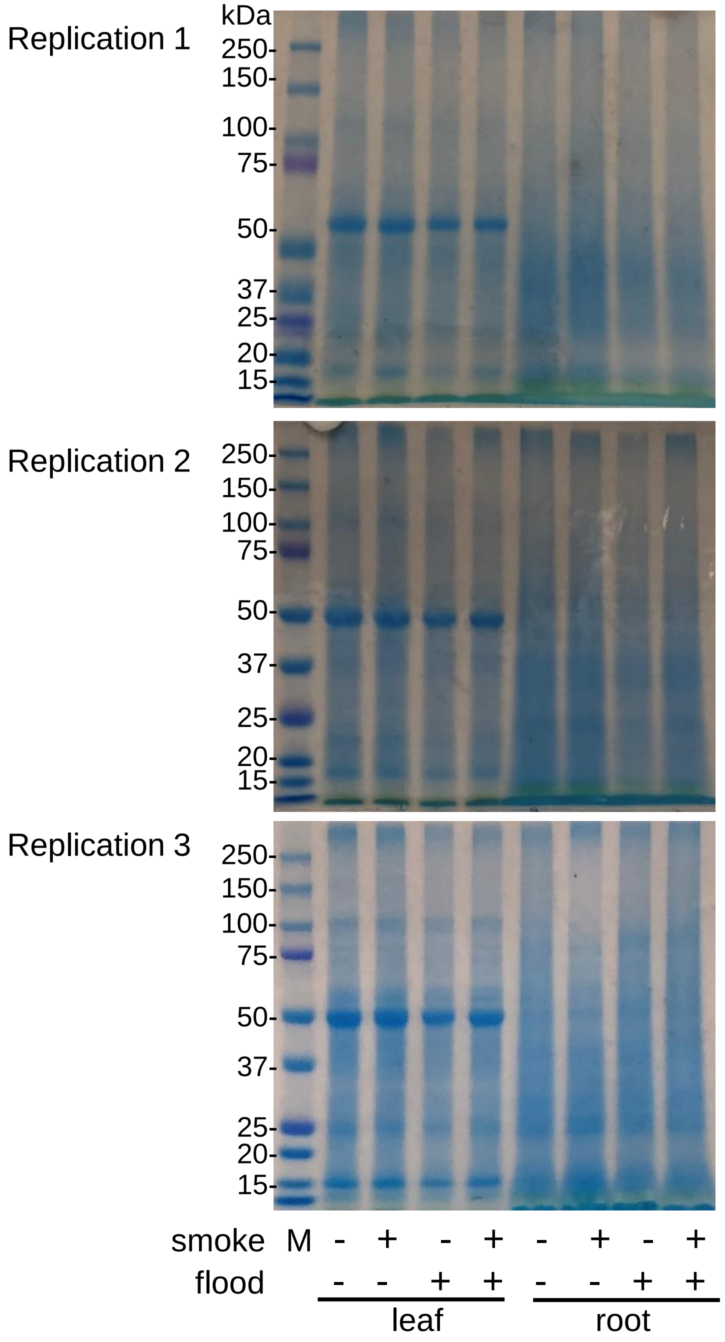

Figure S1. The Coomassie brilliant blue staining patterns of proteins used for immuno-blot analysis. Experiments were performed with biologically triplicates for each treatment. Quantified proteins (10 µg) from leaf and root were separated by electrophoresis on a 10% SDS-polyacrylamide gel. Coomassie brilliant blue staining was used as loading control. “M” means marker proteins.

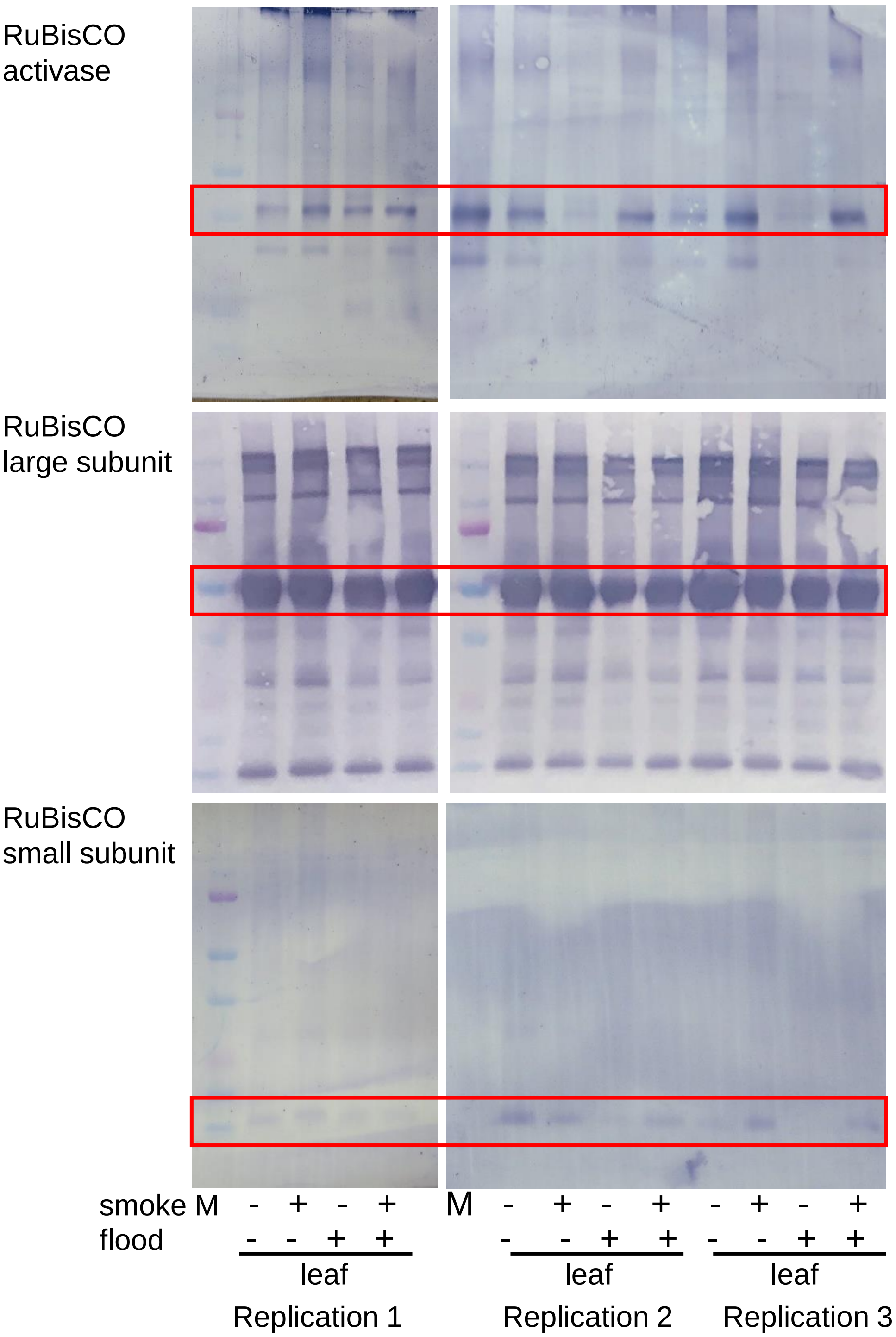

Figure S2. Blots of the entire membrane with anti-RuBisCO activase, RuBisCO large subunit, and RuBisCO small subunit antibodies, which are used in Figure 5. “M” means marker proteins.

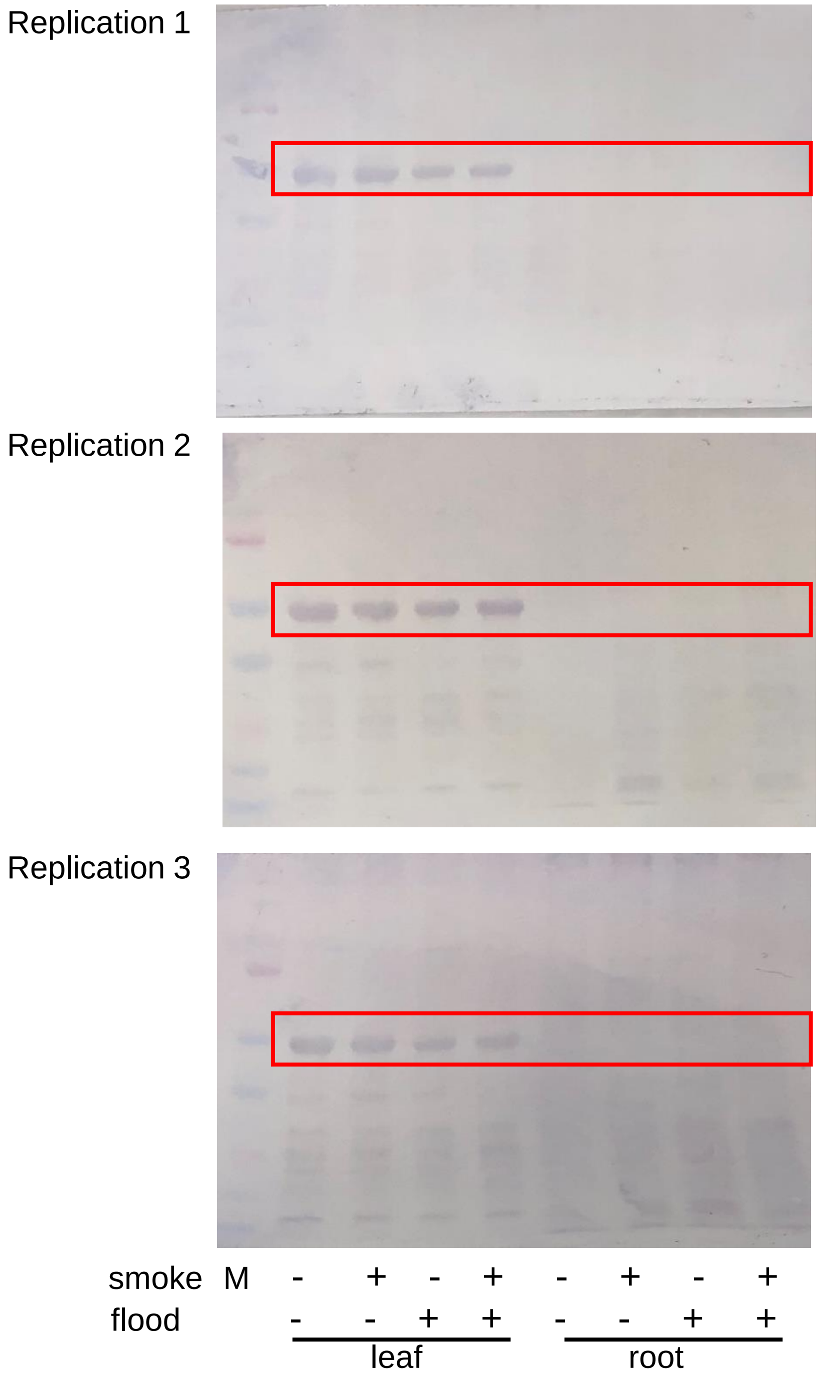

Figure S3. Blots of the entire membrane with anti-FBPA antibody, which are used in Figure 7. “M” means marker proteins.

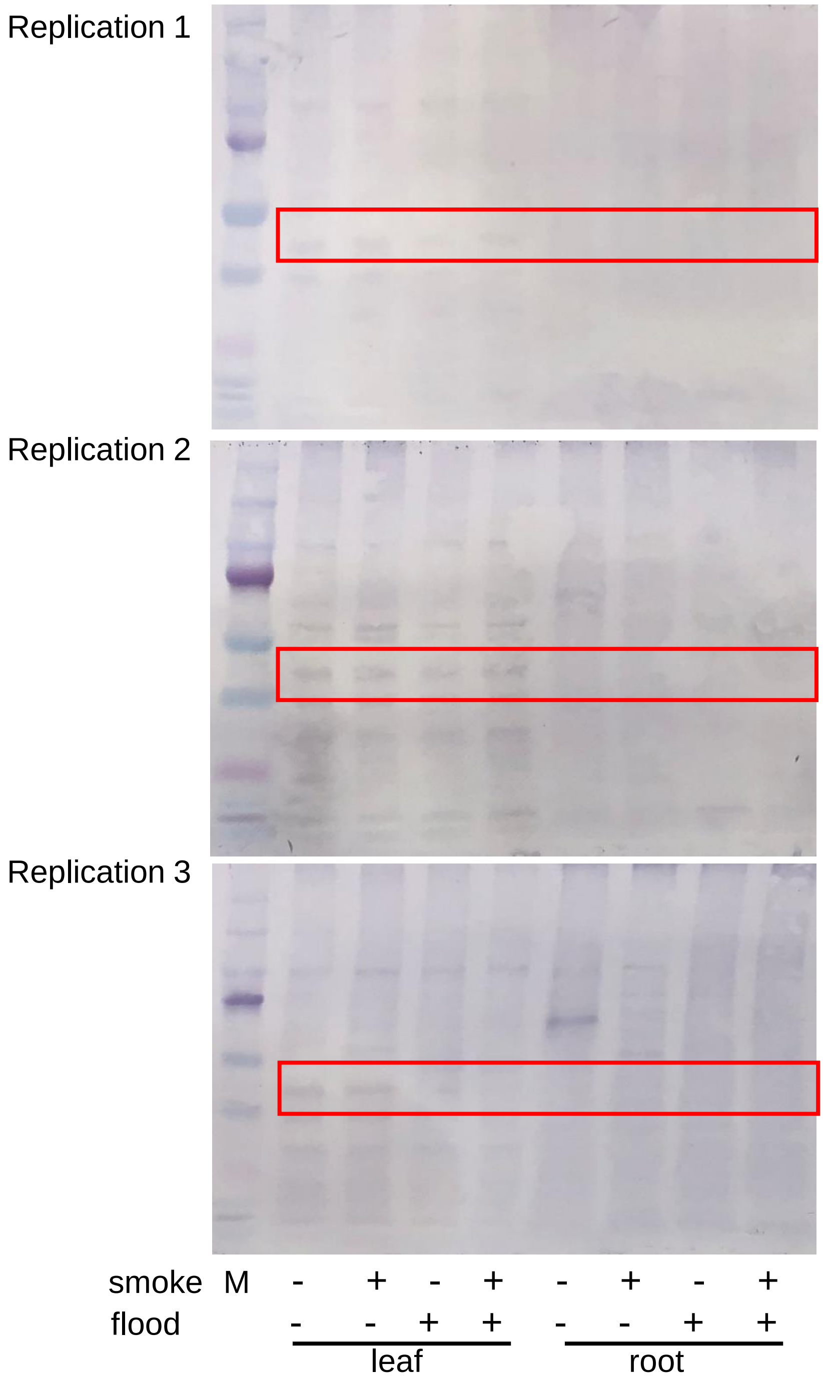

Figure S4. Blots of the entire membrane with anti-TPI antibody, which are used in Figure 7. “M” means marker proteins.

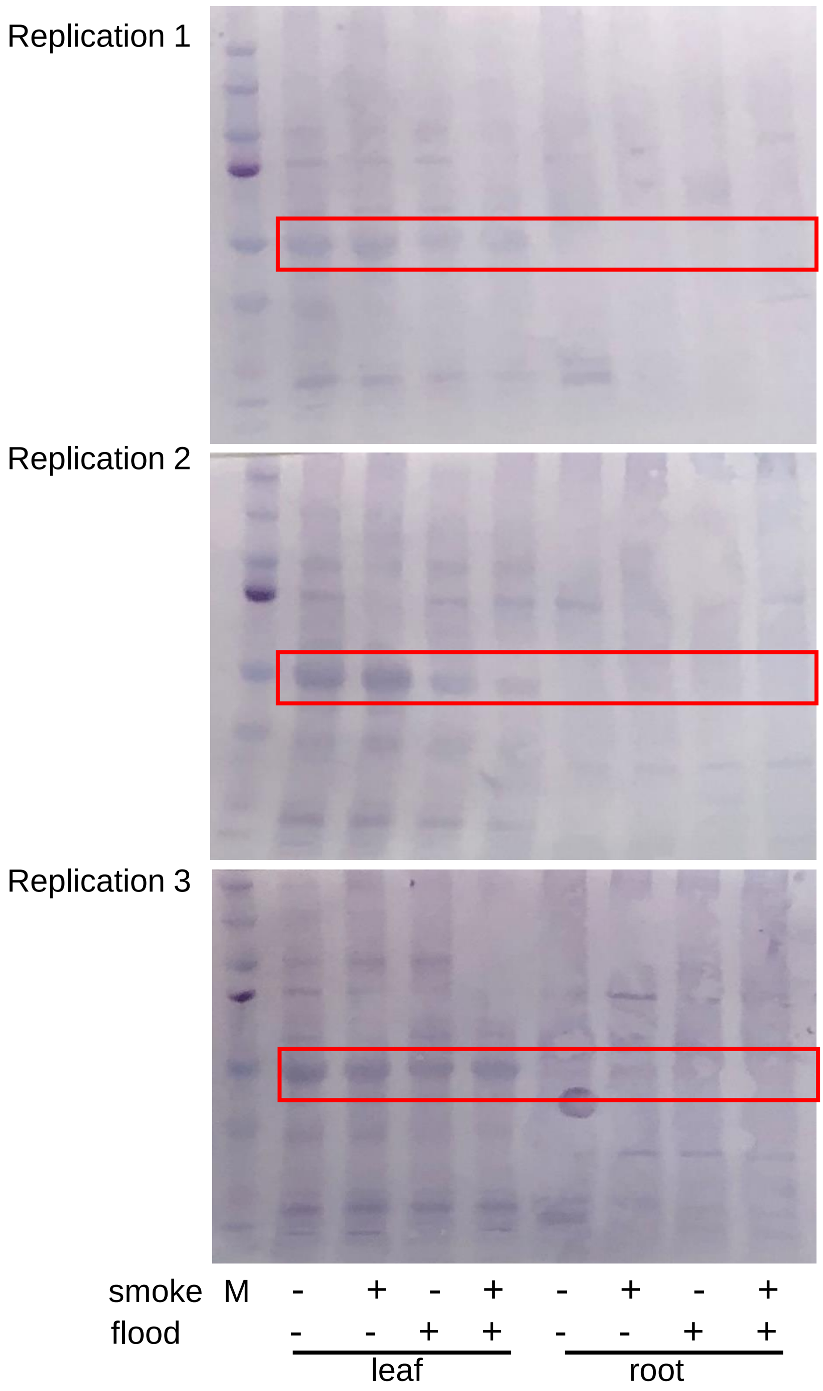

Figure S5. Blots of the entire membrane with anti- GAPDH antibody, which are used in Figure 7. “M” means marker proteins.
