# Supplementary material for: Morphological, Biochemical, and Proteomic Analyses to Understand the Promotive Effects of Plant-Derived Smoke Solution on Wheat Growth under Flooding Stress
Source: Plants (Basel). 2022 Jun 4;11(11):1508. doi: 10.3390/plants11111508 (PMC9183026; doi:10.3390/plants11111508)
Supplement: Supplementary file 1 [file plants-11-01508-s001.zip › plants-1698507-supplementary Tables.pdf]

Table S1. The experimental procedure of gel-free/label-free proteomics used in this research.

Title	Methods
Protein Identification using LC-MS/MS	Peptides were analyzed by LC (EASY-nLC 1000; Thermo Fisher Scientific, San Jose, CA, USA) combined with MS/MS (Orbitrap Fusion ETD MS; Thermo Fisher Scientific) as described in the previous study [46]. The peptides were loaded onto the LC system equipped with a trap column (Acclaim PepMap 100 C18 LC column, 3 µm, 75 µm ID x 20 mm; Thermo Fisher Scientific) equilibrated with 0.1% formic acid and eluted with a linear acetonitrile gradient (0-35%) in 0.1% formic acid at a flow rate of 300 nL/min. The eluted peptides were loaded and separated on the column (EASY-Spray C18 LC column, 3 µm, 75 µm ID x 150 mm; Thermo Fisher Scientific) with a spray voltage of 2 kV (Ion Transfer Tube temperature: 275°C). The peptide ions were detected using MS in the data-dependent acquisition mode with the installed Xcalibur software (version 4.0; Thermo Fisher Scientific). Full-scan mass spectra were acquired in the MS over 375–1,500 m/z, with a resolution of 120,000. The most intense precursor ions were selected for collision-induced fragmentation in the linear ion trap at normalized collision energy of 35%. Dynamic exclusion was employed within 60 sec to prevent the repetitive selection of peptides.
Analysis of MS/MS Data	The MS/MS searches were carried out using the MASCOT (version 2.6.1, Matrix Science, London, U.K.) and SEQUEST HT search algorithms against the UniProtKB *Triticum aestivum* protein database (2017-10-25) using Proteome Discoverer 2.2 (version 2.2.0.388; Thermo Scientific). The condition of the analysis is described in the previous study [12]. The workflow for both algorithms included the spectrum files RC, spectrum selector, MASCOT, SEQUEST HT search nodes, percolator, ptmRS, and minor feature detector nodes. The oxidation of methionine was set as a variable modification, and the carbamidomethylation of cysteine was set as a fixed modification. MS and MS/MS mass tolerances were set to 10 ppm and 0.6 Da, respectively. Trypsin was specified as the protease, and a maximum of one missed cleavage was allowed. Target-decoy database searches were used for the calculation of the false discovery rate, which was set at 1% for peptide identification.
Differential Analysis of Proteins using MS Data	Label-free quantification was performed with Proteome Discoverer 2.2 using precursor ions quantifiler nodes. Principal component analysis (PCA) was also performed with Proteome Discoverer 2.2. For differential analysis of the relative abundance of peptides and proteins between samples, the free software Perseus (version 1.6.2.3, Max Planck Institute of biochemistry, Martinsried, Germany) was used. The condition of analysis is described in the previous study [47]. Protein and peptide abundance was transferred into log2 scale. Three biological replicates of each sample were grouped, and a minimum of three valid values were required in at least one group. Normalization of the abundance was performed to subtract the median of each sample. Missing values were imputed based on a normal distribution (width = 0.3, down-shift = 1.8). Significance was assessed using *t*-test analysis.

Table S2. A list of changed proteins in wheat leaves treated with flooding, compared with the control.

	Difference	Accession	Description	Cov (%)	MP	MW (kDa)	calc pI
1	5.7821	Q5TMB2	Xylanase inhibitor TAXI-IV	22	3	42	8.32
2	5.4650	A0A1D6D7R5	peroxidase	24	2	42.8	7.18
3	4.9763	A0A1D5TIY9	DNA-directed RNA polymerase subunit beta	4	3	136.4	7.34
4	4.7708	A0A1D6CDW2	Dirigent protein	23	2	17.4	5.69
5	4.6527	B5B3P8	Pathogenesis related protein 10	25	2	17.1	5.31
6	4.6403	A9U8G4	Alcohol dehydrogenase ADH1A	37	2	41	6.6

7	4.5883	A0A077RPL6	RuvB-like helicase	6	2	49.7	5.88
8	4.3776	O21432	Ribosomal protein S2	6	2	40.9	9.36
9	4.0711	A7BJ78	xylanase inhibitor	10	2	32.8	8.47
10	4.0322	W5CYT3	Reticulon-like protein	10	2	28.1	8.54
11	3.9330	A0A1D6BD02	rRNA N-glycosidase	17	2	28.3	5.1
12	3.7759	A0A1D5W929	carboxypeptidase	5	2	56.1	6.39
13	3.6866	Q8LK23	peroxidase	9	2	38.8	7.91
14	3.4880	P12119	Cytochrome b6-f complex subunit 4	45	5	17.5	7.18
15	3.4498	P30569	EC protein I/II	56	2	7.7	7.59
16	3.2998	A0A1D5Y4B3	alanine--tRNA ligase	16	2	109.9	6.49
17	3.2552	A0A1D5X8S8	tubulin beta chain	36	2	49.7	4.83
18	3.1417	Q84XZ3	Mitogen-activated protein kinase	8	2	45.3	5.83
19	3.0239	C6ES53	peroxidase	22	3	33	6.19
20	3.0041	A0A1D5VL06	Translation machinery-associated protein 22	22	2	21.3	8.02
21	2.9169	G8HMZ0	myo-inositol-1-phosphate synthase	11	3	56.1	5.92
22	2.8841	W5BCG4	peroxidase	46	5	36.6	6.29
23	2.8239	Q43665	Wali5 protein	40	2	9.5	8.02
24	2.7495	W5I774	sucrose synthase	43	22	92.3	6.09
25	2.6966	A0A1D6RK35	peroxidase	39	2	35.4	7.64
26	2.4972	A0A1D6AQQ7	alpha-amylase	16	4	47.3	6.48
27	2.4517	A0A097KUG3	actin-depolymerizing factor 6	19	2	16.8	5.41
28	2.4054	A0A1D5YTX8	cysteine synthase	37	3	45.3	8.27
29	2.3677	A0A1D5RSR6	Inosine triphosphate pyrophosphatase	13	2	22.7	5.66
30	2.3568	W4ZRX8	ATP-dependent 6-phosphofructokinase	17	5	62.2	7.08
31	2.3157	E0WC53	WALI7	10	2	28.1	7.39
32	2.3130	P01083	Alpha-amylase inhibitor 0.28	19	2	16.8	7.46
33	2.3015	A0A1D5TTP6	peroxidase	37	2	36.6	5.87
34	2.2874	A0A1D5UG55	peroxidase	13	2	36	6.46
35	2.2847	A0A1D5ULY4	Calcium-transporting ATPase	5	5	133.6	6.86
36	2.1587	T1VYS7	Dehydrin WZY1-2	29	2	28.1	5.29
37	2.0208	A0A077RXY9	carboxypeptidase	13	3	51.8	6.62
38	1.9610	A0A1D5VA69	NADH-cytochrome b5 reductase	47	8	31	8.38
39	1.9372	A0A1D6CF61	Polyadenylate-binding protein	14	3	71.7	6.37
40	1.9192	Q8S4P7	Thaumatin-like protein	40	2	23.6	7.61
41	1.8997	A0A1D5SGW0	proline dehydrogenase	6	2	51.2	6.95
42	1.8499	Q6QU77	delta tonoplast intrinsic protein TIP2;3	8	2	25.2	5.69
43	1.7922	A0A172GYI5	PAP fibrillin domain-containing protein	25	2	27.6	10.04
44	1.7504	A0A1D6D8E9	Cytosolic Fe-S cluster assembly factor NBP35	17	2	48.7	5.85
45	1.7053	A0A1D5Z9T5	Lon protease homolog 2, peroxisomal	4	2	97.8	7.91
46	1.6921	A0A1D5Y3Z6	ATP-dependent Clp protease proteolytic subunit	20	4	28.8	6.33
47	1.6664	A0A1D5UML5	peroxidase	40	5	36.4	6.29
48	1.6663	Q5I7L0	ribosomal protein L18	31	4	21.3	11.49
49	1.6213	Q4JH28	beta-1,3-glucanase	34	4	35.3	8.48
50	1.5863	Q8RW01	glutathione transferase	12	2	25.2	5.34
51	1.5818	A0A1D5UUS2	Ribulose bisphosphate carboxylase small chain	54	2	15.3	8.72
52	1.5805	A0A1D5ZI60	Probable bifunctional methylthioribulose-1-phosphate dehydratase/enolase-phosphatase E1	13	2	58.2	6.34
53	1.5666	A0A077KS83	aquaporin	18	4	25.8	6.52
54	1.5312	A0A1D6DEW4	Chlorophyll a-b binding protein, chloroplastic	78	2	27.4	5.25
55	1.5264	A0A1D5UJS1	ferredoxin--NADP reductase	7	3	42.2	8.79
56	1.5077	F2VQK3	Atp4-1	15	3	21.6	9.44

57	1.5006	A0A1D6BI28	Succinate dehydrogenase	20	6	42.1	9.88
58	1.4756	A0A1D6CA75	Acyl-coenzyme A oxidase	9	5	75.6	8.06
59	1.4504	A0A1D5SSP1	Chlorophyll a-b binding protein, chloroplastic	94	2	16.9	4.88
60	1.4448	A0A1D5TG44	ammonium transporter	7	2	52.2	7.49
61	1.4346	W5DVM8	3-ketoacyl-CoA synthase	5	2	54.2	8.65
62	1.4007	A0A1D6CPZ3	peroxidase	40	2	34.2	8.73
63	1.3809	A0A1D6AMT2	Ubiquinol oxidase	15	5	47.5	9.83
64	1.3415	A0A077S025	Protein ROOT HAIR DEFECTIVE 3 homolog	14	7	90.2	5.54
65	1.3350	W5FWT6	aldehyde dehydrogenase 7B1	32	10	54.3	6.44
66	1.3207	B2BA42	V-type proton ATPase subunit	28	6	40.7	5
67	1.3164	A0A1D5XC94	Histone H2A	46	2	17.9	10.78
68	1.3159	A0A1D5SEA5	Eukaryotic translation initiation factor 3 subunit D	22	7	65.7	5.72
69	1.2954	A0A1D5WG52	H/ACA ribonucleoprotein complex subunit	13	2	23.4	11.47
70	1.2741	A0A1D5YQZ4	peptidyl-prolyl cis-trans isomerase	23	2	15.5	9.35
71	1.2653	W5H631	60S ribosomal protein L27	39	2	15.5	10.48
72	1.2508	A0A1D5SX23	FRIGIDA-like protein	9	3	68	8.28
73	1.2406	Q5I5K7	small GTP-binding protein	36	2	22.9	7.36
74	1.2395	A0A1D6RP57	sucrose synthase	3	2	99	8.29
75	1.2372	W4ZQS8	Mitogen-activated protein kinase	20	5	42.8	6.16
76	1.2310	A0A1D6CXT4	Proteasome subunit alpha type	32	4	26.9	6.79
77	1.2301	A0A1D6BGW9	Delta-aminolevulinic acid dehydratase	40	6	49.5	7.83
78	1.2126	Q1XIR9	4-hydroxy-7-methoxy-3-oxo-3,4-dihydro-2H-1,4-benzoxazin-2-yl glucoside beta-D-glucosidase 1a, chloroplastic	60	9	64.5	7.02
79	1.2124	A0A024FRN8	Protein disulfide isomerase family protein 5-1	46	3	47.2	5.4
80	1.2071	A0A1D5SBJ1	signal recognition particle 54 kDa protein	6	2	54.5	9.29
81	1.1970	A0A1D5U0D2	Ubiquinol oxidase	8	2	38.3	5.59
82	1.1893	D3JUT3	xylanase inhibitor protein I	29	8	33.3	8.47
83	1.1843	A0A1D5SAE3	60S ribosomal protein L18a	37	2	21.2	10.35
84	1.1697	Q7XY22	Mitochondrial pyruvate carrier	42	4	12.3	9.5
85	1.1683	A0A1D5SY80	60S ribosomal protein L36	15	2	14.6	11.11
86	1.1591	A0A1D5X8I7	pyruvate kinase	19	6	57.8	6.33
87	1.1469	A0A1D6S7W6	auxin efflux carrier component	5	2	73.8	9.67
88	1.1384	B4F6E6	peroxidase	50	3	32.5	8.06
89	1.1257	A0A1D5U5D9	amine oxidase	8	3	85.7	7.08
90	1.1221	A0A1D5VHR2	Reticulon-like protein	20	4	35	7.3
91	1.1071	A0A1D5UXT7	cysteine proteinase inhibitor	54	5	13.1	10.13
92	1.1060	A0A1D5T1L6	methionine S-methyltransferase	6	3	122.4	5.53
93	1.0888	M4PPG8	glycerol kinase	11	4	57.2	5.63
94	1.0800	C0LF30	Serpin 1	20	2	43.1	5.69
95	1.0758	A0A1D5YN81	Polyadenylate-binding protein	27	9	70.7	7.37
96	1.0739	Q45NB5	Glutamine synthetase	47	9	39.2	5.6
97	1.0713	P58386	Photosystem I P700 chlorophyll a apoprotein A2	33	14	82.5	7.15
98	1.0669	A0A1D6AA00	Serine/threonine-protein phosphatase	8	2	43.5	6.19
99	1.0525	W5DXL5	Eukaryotic translation initiation factor 3 subunit K	35	5	25.9	5.16
100	1.0275	A0A1D5SFV9	histone H4	16	8	34.5	10.05
101	1.0249	W5F8V5	Eukaryotic translation initiation factor 3 subunit E	36	9	50.7	5.69
102	1.0063	A0A1D5U388	proliferating cell nuclear antigen	45	8	30.8	4.84
103	1.0021	A0A1D6RS59	Protein YIPF	14	3	34.2	7.53
104	0.9880	W5FZV5	Annexin	22	5	35.4	8.87
105	0.9751	B2CGM6	triticin	10	3	64.9	6.92

106	0.9707	A0A1D6CY42	peptidylprolyl isomerase	24	10	61.9	5.4
107	0.9685	I7KM78	Gamma-gliadin OS=Triticum aestivum GN=GID-HE1 PE=2 SV=1	8	2	38	7.24
108	0.9663	A0A1D5UAF0	Chlorophyll a-b binding protein, chloroplastic	64	2	17.1	5.5
109	0.9608	Q6V959	ribosomal protein L3	33	7	44.6	10.07
110	0.9571	P17314	Alpha-amylase/trypsin inhibitor CM3	42	4	18.2	7.44
111	0.9548	A0A075VVI2	50S ribosomal protein L22, chloroplastic	18	2	18.2	10.93
112	0.9431	Q75QN8	cold shock domain protein 3	55	3	21.5	6.1
113	0.9158	A0A1D5XSR2	Xyloglucan endotransglucosylase/hydrolase	21	5	33.5	7.01
114	0.9014	A0A1D6BGI5	Protein arginine N-methyltransferase	6	2	73.6	5.39
115	0.8994	P52589	Protein disulfide-isomerase	43	4	56.5	5.1
116	0.8979	Q95H56	50S ribosomal protein L33, chloroplastic	32	2	7.7	9.94
117	0.8973	A0A1D5UX72	glycosyltransferase	12	2	53.6	6.09
118	0.8898	A0A1D5YUF6	Probable bifunctional methylthioribulose-1-phosphate dehydratase/enolase-phosphatase E1	13	2	56.7	6.44
119	0.8867	Q03387	Eukaryotic translation initiation factor isoform 4G-1	28	3	86.2	8.02
120	0.8724	Q5EWZ1	Actin	62	2	41.7	5.4
121	0.8723	A0A1D6DKV5	Inositol-tetrakisphosphate 1-kinase	11	2	36.7	5.16
122	0.8719	P60140	Photosystem II reaction center protein L	100	3	4.5	4.5
123	0.8692	C1K737	multiprotein bridging factor 1	32	4	15.7	9.88
124	0.8649	A0A1D5W682	cysteine synthase	13	3	56.8	8.34
125	0.8640	A7L5U5	Mitogen-activated protein kinase	8	2	42.8	5.76
126	0.8566	A0A1D5YCN6	Mitochondrial Rho GTPase	22	9	71.3	5.91
127	0.8499	P69415	Photosystem I iron-sulfur center	64	5	8.9	6.92
128	0.8419	W5D6S5	S-formylglutathione hydrolase	37	7	31.6	6.61
129	0.8408	A0A1D6CUB4	Histone H2A	44	2	25.3	10.96
130	0.8281	A0A1D6RW56	Nuclear pore protein	3	2	95.9	6.47
131	0.8191	L0N608	cell wall invertase	20	7	64.5	8.28
132	0.8153	A0A1D5UJG2	protoporphyrinogen oxidase	12	3	58.9	9.19
133	0.8099	A0A1D5YSI2	Transmembrane 9 superfamily member	10	2	67.6	8.65
134	0.8016	C6K7G3	Lipoxygenase	48	2	96.5	6.65
135	0.7920	Q41518	single-stranded nucleic acid binding protein	72	4	16.2	5.25
136	0.7903	A0A1D5UMU8	plasma membrane ATPase	43	25	104.7	6.81
137	0.7897	A0A1D6CBF2	peroxidase	23	5	36.8	9.51
138	0.7831	A0A1D5UR98	Anamorsin homolog	15	2	27.7	4.89
139	0.7815	L7PE91	Farnesyl pyrophosphate synthase	22	4	40.5	5.06
140	0.7810	F4Y590	heat shock protein 90	21	9	81.2	5.05
141	0.7770	Q3S861	Pyridoxine biosynthesis protein	24	4	33.3	6.87
142	0.7739	W5EC46	Non-specific lipid-transfer protein	36	3	12.2	9.06
143	0.7664	Q7XYE0	NADPH-dependent mannose 6-phosphate reductase	29	2	30.1	6.9
144	0.7562	A0A1D6CMR8	peptidyl-prolyl cis-trans isomerase	28	5	23.4	9.57
145	0.7499	A0A1D5Z0H9	Proteasome subunit beta type	51	9	27.8	7.27
146	0.7457	W5DZ64	Acyl carrier protein	17	2	14.6	5.94
147	0.7386	A0A1D6BNE8	Proteasome subunit beta type	24	2	22.9	5.44
148	0.7193	A0A1D6AZU5	peroxidase	62	3	34.5	7.83
149	0.7173	A0A1D5X3V0	PRA1 family protein	13	2	23.3	8.02
150	0.7141	A0A1D6SBV0	T-complex protein 1 subunit gamma	31	12	60.9	6.33
151	0.7060	A0A1D5UYD6	Phosphoinositide phospholipase C	7	3	68.4	6.3
152	0.7047	A0A1D5S627	peroxidase	7	2	41.9	7.64
153	0.7022	A0A1D5RTN9	proline iminopeptidase	10	2	44.4	7.85
154	0.7005	W5FB88	Chlorophyll a-b binding protein, chloroplastic	77	2	28.3	5.43

155	0.6938	A0A1D5VDW6	aspartate aminotransferase	37	12	49.6	8.47
156	0.6920	A0A1D5Z7E3	Serine/threonine-protein phosphatase	13	2	35.2	5.33
157	0.6634	I3NM23	Lipoxygenase	42	12	95.9	6.28
158	0.6625	C8CK09	phosphomannomutase	11	2	28.4	6.04
159	0.6615	A0A1D5UBN2	UMP-CMP kinase	36	7	27.2	5.8
160	0.6578	W5GDZ8	4-hydroxyphenylpyruvate dioxygenase	17	5	46.3	5.78
161	0.6398	O80370	Dirigent protein	59	11	32.4	7.18
162	0.6341	A0A1D5WD63	Pyrroline-5-carboxylate reductase	15	3	29.5	7.99
163	0.6314	A0A1D5X3A9	Eukaryotic translation initiation factor 3 subunit I	27	8	42.6	8.25
164	0.6301	C7C4X1	glyceraldehyde-3-phosphate dehydrogenase	54	7	36.5	7.18
165	0.6296	A0A1D5RU17	guanosine nucleotide diphosphate dissociation inhibitor	27	5	55.6	5.57
166	0.6280	A0A1D5ZUF5	inosine-5'-monophosphate dehydrogenase	21	2	52.1	6.06
167	0.6265	A5HE90	Hypersensitive response protein	21	5	31.3	5.44
168	0.6128	P04464	Calmodulin	53	2	16.8	4.25
169	0.6116	F1DKC1	catalase	66	19	56.8	7.08
170	0.6021	A0A1D5Y0P8	Translocase of chloroplast	20	5	36.3	9.35
171	0.6006	W5F8S6	Succinate dehydrogenase	26	10	68.2	6.57
172	0.5981	A0A1D5YRC9	Chlorophyll a-b binding protein, chloroplastic	78	2	28.3	5.43
173	0.5979	Q8RW00	glutathione transferase	34	6	26.4	5.1
1	-0.5928	W5GWD7	peptidylprolyl isomerase	17	2	20.4	9.26
2	-0.5945	A0A1D6BUR5	peptidylprolyl isomerase	28	4	24.8	7.55
3	-0.5972	A6Q0N9	putative acyl transferase 4	34	2	46.4	6.05
4	-0.5980	A0A077RW30	cysteine synthase	59	5	41.1	6.4
5	-0.5996	W5AMW4	peroxidase	15	3	35.9	8.27
6	-0.6031	A0A1D5VGQ8	NADH-cytochrome b5 reductase	36	8	33.8	8.51
7	-0.6193	W5G255	MBD6-5DS	65	10	43.6	4.54
8	-0.6312	A0A1D6CMU2	Beta-galactosidase	22	3	78.5	8.1
9	-0.6347	A0A1D5SYC3	phosphotransferase	8	2	55.2	6.73
10	-0.6384	A0A1D5UA20	Lon protease homolog, mitochondrial	9	5	107.4	5.58
11	-0.6432	W5DEV0	peptidylprolyl isomerase	26	3	16.5	8.25
12	-0.6445	A0A1D5VSF8	pectin acetylesterase	6	2	47.8	6.43
13	-0.6469	A0A1D5XKY8	coatomer subunit alpha	28	2	135.7	7.01
14	-0.6476	A0A1D5X268	ferredoxin--NADP reductase	52	5	39.9	8.25
15	-0.6525	A0A1D6BR25	peroxidase	24	5	39.3	6.96
16	-0.6586	A0A1D6AB44	Lysine--tRNA ligase	9	4	65.5	6.51
17	-0.6596	A0A1D6D779	Threonine dehydratase	4	2	64.9	6.04
18	-0.6680	A0A1D5ZLG5	Beta-galactosidase	12	6	101.2	5.59
19	-0.6689	Q5NE29	Non-specific lipid-transfer protein	36	2	12.4	9.23
20	-0.6725	A0A1D5YGQ4	glucose-6-phosphate 1-epimerase	39	2	34.4	6.09
21	-0.6769	A0A1D6BMD7	Superoxide dismutase	32	3	20.3	5.45
22	-0.6836	A0A1D5YI87	UDP-glucose 6-dehydrogenase	46	2	52.6	6.35
23	-0.6843	A1YUM8	Limit dextrinase type starch debranching enzyme	11	6	106	5.91
24	-0.6886	A0A1D6D5W3	Probable alanine--tRNA ligase, chloroplastic	27	4	106.9	6.27
25	-0.7004	W5AL94	peroxidase	32	7	36.3	6.15
26	-0.7025	A0A1D6CV93	peroxidase	22	4	38.4	4.86
27	-0.7107	A0A1D5YP74	Mg-protoporphyrin IX chelatase	27	15	82.6	5.58
28	-0.7125	Q401N6	aspartic proteinase	19	3	54	6.15
29	-0.7152	A0A1D5ZTH5	Chlorophyll a-b binding protein, chloroplastic	56	2	28.3	5.43
30	-0.7212	W5E5Z3	ribulose-phosphate 3-epimerase	47	5	28.9	8.15
31	-0.7238	C1IP75	lycopene epsilon cyclase 3B	4	2	59.6	7.31

32	-0.7340	A0A1D5TCZ2	pectin acetylesterase	16	5	42.6	7.66
33	-0.7376	A0A1D6CUI4	glycosyltransferase	8	2	51.2	5.62
34	-0.7398	Q9S8J6	Basic protein 3	100	2	2.1	7.99
35	-0.7448	P69555	Photosystem II reaction center protein H	23	2	7.8	8.57
36	-0.7453	A0A1D6BQY9	tubulin beta chain	56	4	50.2	4.81
37	-0.7554	W5QKZ0	Chalcone-flavonone isomerase family protein	68	3	23.6	5.12
38	-0.7618	A0A1D6RQH6	pyruvate kinase	23	6	59	7.25
39	-0.7618	A0A1D5YPG2	malate dehydrogenase	60	9	37.2	8.18
40	-0.7707	A0A1D5TDS2	acetyl-coenzyme A synthetase	7	2	80.8	6.54
41	-0.7727	A0A1D5STK8	glycosyltransferase	33	4	50.6	5.35
42	-0.7741	D3K4D8	ATP synthase subunit	57	3	39.7	8.02
43	-0.7798	W5CQ97	cysteine proteinase inhibitor	39	2	26.7	6.87
44	-0.7928	W5QKY9	Chalcone-flavonone isomerase family protein	54	2	23.7	4.93
45	-0.7931	A0A1D5WDB9	Diphosphomevalonate decarboxylase	7	2	46.1	6.93
46	-0.7947	A0A1D5SBK9	malate dehydrogenase	70	2	35.5	8.38
47	-0.7954	Q8L686	adenosine diphosphate glucose pyrophosphatase	36	3	21.8	6.13
48	-0.8023	W5FL28	Lipoxygenase	42	6	105.4	6.11
49	-0.8023	Q75RZ2	Putative caffeoyl CoA O-methyltransferase	13	3	29.1	5.21
50	-0.8054	A0A1D5UQS0	peroxidase	18	2	26.8	8.59
51	-0.8286	A0A1D6BHG2	Transmembrane 9 superfamily member	5	2	71.8	6.47
52	-0.8326	A0A1D5UTK1	GrpE protein homolog	25	4	37.2	7.4
53	-0.8446	Q56TP1	Xyloglucan endotransglucosylase/hydrolase	22	2	32.3	6.05
54	-0.8456	A0A1D5UP29	Lipoxygenase	33	5	101.8	6.43
55	-0.8579	A0A1D6B9G2	Phenylalanine ammonia-lyase	57	23	80.4	6.49
56	-0.8671	Q6EZE8	sucrose-phosphate synthase	17	2	115.1	6.2
57	-0.8679	A0A1D6DD56	alpha-amylase	7	4	97.4	5.76
58	-0.8685	D3KVP3	Purple acid phosphatase	10	2	37.9	5.86
59	-0.8934	W5G4K3	Lipoxygenase	46	3	105.3	6.1
60	-0.9081	A0A172WCB1	cold-responsive LEA/RAB-related COR protein	24	2	17.2	4.96
61	-0.9124	A0A1D6RZ25	UDP-glucose 6-dehydrogenase	43	3	40.8	6.34
62	-0.9144	A0A1D5SQI2	Nucleoside diphosphate kinase	16	4	35.8	9.31
63	-0.9194	A0A1D6BFL6	glycosyltransferase	16	2	51.6	5.82
64	-0.9329	A0A1D5WPC9	glycine cleavage system P protein	76	3	99.5	6.43
65	-0.9637	C0KTA6	fructose-bisphosphate aldolase	76	2	42	6.16
66	-0.9866	A0A1D5ZXR0	tubulin alpha chain	43	2	49.7	4.98
67	-1.0012	W5BBF4	Lipoxygenase	41	10	101.8	6.35
68	-1.0130	Q9XPT0	ATP synthase subunit a, chloroplastic	37	5	27.3	5.47
69	-1.0170	A0A1D5UXB4	pectin acetylesterase	46	10	45.1	8.38
70	-1.0172	A0A1D6CVC4	Ferredoxin	64	4	15.3	4.68
71	-1.0173	A0A1D5U2X7	Glutathione peroxidase	15	2	19.3	7.84
72	-1.0183	A0A1D5RYL8	Lipoxygenase	8	4	94.9	7.62
73	-1.0355	A0A1D5SR02	carboxypeptidase	14	4	48.1	7.18
74	-1.0471	Q9LDX4	thioredoxin	69	2	13.3	5.22
75	-1.0651	A0A1D5XE21	phosphoserine aminotransferase	27	3	42.4	8.85
76	-1.0676	W5AAU0	Chalcone-flavonone isomerase family protein	11	2	28.8	9.42
77	-1.0691	A0A1D5XJV3	tubulin beta chain	57	2	50.2	4.79
78	-1.0798	A0A077S1E6	pyruvate kinase	16	2	63	6.4
79	-1.0849	W5ASP5	Lipoxygenase	32	6	101.4	6.28
80	-1.1016	P29557	Eukaryotic translation initiation factor 4E-1	22	3	24	5.62

81	-1.1151	A0A1D6S8Q3	Lipoxygenase	27	13	102.3	6.14
82	-1.1297	Q2PCB9	Non-specific lipid-transfer protein	38	3	11.6	8.69
83	-1.1454	W5AGK9	Nucleoside diphosphate kinase	70	2	16.7	6.8
84	-1.1457	A0A1D5SHA7	D-3-phosphoglycerate dehydrogenase	25	8	64.7	8.44
85	-1.1509	W5B7N4	Eukaryotic translation initiation factor 3 subunit H	19	6	38.8	4.92
86	-1.1557	A0A1D5YYQ8	Dirigent protein	62	3	12.3	6.7
87	-1.1588	Q0WXG6	Triticain gamma	38	3	39.4	6.84
88	-1.1637	A0A0C4BK97	glycine cleavage system H protein	56	2	17.3	5.15
89	-1.1746	A0A0C4BK59	glycine cleavage system H protein	68	3	17.3	5.15
90	-1.1783	A0A1D5RZ51	beta-hexosaminidase	21	2	61.9	6.62
91	-1.1888	A0A1D6C4A1	Thiamine thiazole synthase, chloroplastic	37	9	42.6	6.24
92	-1.2126	A0A1D5YMV3	pectin acetylesterase	28	6	41.9	7.75
93	-1.2184	Q4Z8L8	class II chitinase	39	2	28.2	8.37
94	-1.2214	A0A1D5T678	Clathrin light chain	11	2	33.2	4.86
95	-1.2331	A0A1D5YEQ8	Dirigent protein	44	3	17.1	6.79
96	-1.2663	A0A1D6DCN8	acylphosphatase	18	2	13.6	11.3
97	-1.2677	W5ESB9	Endoglucanase	12	5	69.7	8.65
98	-1.2699	A0A1D6CWU9	hexosyltransferase	6	2	58.8	9.45
99	-1.2817	A0A1D6ADS5	Protein disulfide-isomerase	3	2	67.3	4.75
100	-1.2918	W5GZC2	Transmembrane 9 superfamily member	4	2	74.2	6.79
101	-1.2924	A0A1D6AYW9	Lipoxygenase	4	2	99.4	6.32
102	-1.3757	A0A1D5TC74	peroxidase	23	4	34.2	4.84
103	-1.3813	A0A1D6RDG1	PRA1 family protein	11	2	23.2	9.04
104	-1.3815	A0A1D5YU28	Elongation factor Ts, mitochondrial	45	6	91	4.87
105	-1.4023	A0A1D5V2T1	Cellulose synthase	12	7	123.1	7.24
106	-1.4414	F8WTQ7	Delta-aminolevulinic acid dehydratase	59	3	46.2	5.83
107	-1.4497	A0A1D5W8L4	Dirigent protein	44	2	17.3	6.68
108	-1.4805	A0A1D6BSN8	plastocyanin	46	2	15.6	5.87
109	-1.5200	A0A1D5XG45	Purple acid phosphatase	37	4	69.1	6.38
110	-1.5539	W5G3J1	Lipoxygenase	12	6	107.6	6.35
111	-1.5592	Q1KMV1	Non-specific lipid-transfer protein	37	3	11.2	9.23
112	-1.5850	Q84N29	Probable non-specific lipid-transfer protein 3	36	2	12.3	9.42
113	-1.5928	A0A077RVJ9	Non-specific lipid-transfer protein	37	2	11.1	8.87
114	-1.6324	A0A1D6A1A8	Acyl carrier protein	11	2	15.9	5.6
115	-1.6352	Q8W431	Sucrose:fructan 6-fructosyltransferase	37	3	68.4	5.5
116	-1.6690	A0A1D6CIV2	glycosyltransferase	5	2	49.5	5.6
117	-1.6774	A0A1D5UG33	Non-specific lipid-transfer protein	24	2	18.4	9.42
118	-1.6871	W5EXB7	Fatty acyl-CoA reductase	15	5	55.6	8.09
119	-1.7223	A0A1D6C1S7	Elongation factor Ts, mitochondrial	7	2	41.3	8.28
120	-1.7929	A0A1D6BLG4	aspartate aminotransferase	23	2	63	8.02
121	-1.8256	A0A1D5WVE3	Non-specific lipid-transfer protein	21	2	20.2	9.19
122	-1.8486	A0A1D6D5D4	plastocyanin	46	2	15.6	5.87
123	-1.8817	A0A1D5VMR3	Dirigent protein	50	2	17.3	6.37
124	-1.9378	O04437	glutathione S-transferase	15	2	23.7	6.52
125	-1.9757	W5FWD9	peroxidase	11	2	39	8.75
126	-2.0465	A0A1D5V7B0	pyruvate kinase	16	2	62.9	6.37
127	-2.0966	A0A1D5Z579	Cellulose synthase	8	5	124.5	7.85
128	-2.1899	A0A0R6UPC9	CSLF6	3	2	105	8.37
129	-2.2117	D8L9L4	BolA-like protein domain containing protein,expressed	45	2	10.1	5.78

130	-2.3392	A0A1D6DFK9	beta-amylase	61	2	56.6	5.43
131	-2.3595	G9HXG8	Isoamylase 3	9	4	86.2	6.54
132	-2.4490	A0A1D5UKS5	gamma-glutamylcyclotransferase	19	3	23.9	5.43
133	-2.4639	A0A1D6D1F6	sucrose synthase	13	3	92.6	6.27
134	-2.4780	A0A125RLL7	MBD6-5BS	57	5	46.3	4.5
135	-2.8172	A0A1D6CMN4	glutathione synthetase	8	3	62.8	6.27
136	-3.2632	A0A1D6BWT2	Rhomboid-like protein	9	2	35.7	8.66
137	-3.5352	W5DQW1	peroxidase	12	2	34.4	5.77
138	-3.5830	A0A1D5VSY7	peroxidase	14	3	37.5	8.47
139	-3.7256	A0A1D5SI76	Xyloglucan endotransglucosylase/hydrolase	8	2	31	4.94
140	-4.2942	A0A1D6DBP8	beta-amylase	13	5	72.1	6.04
141	-4.3942	A0A0H3WFR0	Glycerol-3-phosphate acyltransferase 6	5	2	55.3	9.17

Cov, coverage; MP, matched peptides; MW, molecular weight; and calc pI, calculated pI.

Table S3. A list of the changed proteins in wheat leaves treated with flooding and plant-derived smoke solution, compared with the control.

	Difference	Accession	Description	Cov (%)	MP	MW (kDa)	calc pI
1	6.5202	A0A1D6CDW2	Dirigent protein	23	2	17.4	5.69
2	6.0195	P01083	Alpha-amylase inhibitor 0.28	19	2	16.8	7.46
3	4.7260	A0A1D6AQQ7	alpha-amylase	16	4	47.3	6.48
4	4.5325	A0A1D5TIY9	DNA-directed RNA polymerase subunit beta	4	3	136.4	7.34
5	4.3452	A0A1D5Y4B3	alanine--tRNA ligase	16	2	109.9	6.49
6	3.7563	P12119	Cytochrome b6-f complex subunit 4	45	5	17.5	7.18
7	3.6764	A0A077RPL6	RuvB-like helicase	6	2	49.7	5.88
8	3.6762	A0A1D5UUS2	Ribulose bisphosphate carboxylase small chain	54	2	15.3	8.72
9	3.4629	W5CYT3	Reticulon-like protein	10	2	28.1	8.54
10	3.4338	Q8S4P7	Thaumatin-like protein	40	2	23.6	7.61
11	3.3369	A0A1D5W929	carboxypeptidase	5	2	56.1	6.39
12	2.9630	A0A1D6RP57	sucrose synthase	3	2	99	8.29
13	2.8944	A0A1D6RW56	Nuclear pore protein	3	2	95.9	6.47
14	2.8856	A0A1D5YTX8	cysteine synthase	37	3	45.3	8.27
15	2.8784	Q84XZ3	Mitogen-activated protein kinase	8	2	45.3	5.83
16	2.8237	A0A1D5SGW0	proline dehydrogenase	6	2	51.2	6.95
17	2.7086	Q8LK23	peroxidase	9	2	38.8	7.91
18	2.7036	A0A1D5X8S8	tubulin beta chain	36	2	49.7	4.83
19	2.6831	A0A097KUG3	actin-depolymerizing factor 6	19	2	16.8	5.41
20	2.6546	T1VYS7	Dehydrin WZY1-2	29	2	28.1	5.29
21	2.5628	A0A1D5RSR6	Inosine triphosphate pyrophosphatase	13	2	22.7	5.66
22	2.5005	A0A1D5ULY4	Calcium-transporting ATPase	5	5	133.6	6.86
23	2.4458	A0A1D5Y3Z6	ATP-dependent Clp protease proteolytic subunit	20	4	28.8	6.33
24	2.4085	G8HMZ0	myo-inositol-1-phosphate synthase	11	3	56.1	5.92
25	2.3721	A0A077RXY9	carboxypeptidase	13	3	51.8	6.62
26	2.2752	A0A1D5XGF3	beta-amylase	37	3	58.7	5.58
27	2.2433	C6ES53	peroxidase	22	3	33	6.19
28	2.1391	A0A1D5XSR2	Xyloglucan endotransglucosylase/hydrolase	21	5	33.5	7.01
29	2.0389	B2CGM6	triticin	10	3	64.9	6.92
30	2.0124	A0A1D6B7C8	Phenylalanine ammonia-lyase	24	8	77.1	6.19
31	1.9895	A0A1D6CPZ3	peroxidase	40	2	34.2	8.73
32	1.9796	W4ZRX8	ATP-dependent 6-phosphofructokinase	17	5	62.2	7.08
33	1.9173	P30569	EC protein I/II	56	2	7.7	7.59
34	1.9046	W5DVM8	3-ketoacyl-CoA synthase	5	2	54.2	8.65
35	1.8664	P17314	Alpha-amylase/trypsin inhibitor CM3	42	4	18.2	7.44
36	1.8288	Q2PCB9	Non-specific lipid-transfer protein	38	3	11.6	8.69
37	1.8149	A0A1D6RK35	peroxidase	39	2	35.4	7.64
38	1.8094	A0A1D5ZI60	Probable bifunctional methylthioribulose-1-phosphate dehydratase/enolase-phosphatase E1	13	2	58.2	6.34
39	1.7868	A0A1D6CF61	Polyadenylate-binding protein	14	3	71.7	6.37
40	1.7435	A0A1D5UJS1	ferredoxin--NADP reductase	7	3	42.2	8.79
41	1.7365	A0A1D5VL06	Translation machinery-associated protein 22	22	2	21.3	8.02
42	1.7242	A0A1D6CXT4	Proteasome subunit alpha type	32	4	26.9	6.79
43	1.6968	A0A1D5X8I7	pyruvate kinase	19	6	57.8	6.33
44	1.6860	C0LF30	Serpin 1	20	2	43.1	5.69
45	1.6841	A0A1D5YQZ4	peptidyl-prolyl cis-trans isomerase	23	2	15.5	9.35
46	1.6551	A0A1D6BI28	Succinate dehydrogenase	20	6	42.1	9.88

47	1.6468	A0A1D5T329	Alpha-mannosidase	14	7	83.8	6.77
48	1.6057	A0A1D5UXB4	pectin acetylesterase	46	10	45.1	8.38
49	1.5799	W5EC46	Non-specific lipid-transfer protein	36	3	12.2	9.06
50	1.5594	P60140	Photosystem II reaction center protein L	100	3	4.5	4.5
51	1.5388	Q5I7L0	ribosomal protein L18	31	4	21.3	11.49
52	1.5022	A0A1D5X3V0	PRA1 family protein	13	2	23.3	8.02
53	1.4462	A0A1D6D8E9	Cytosolic Fe-S cluster assembly factor NBP35	17	2	48.7	5.85
54	1.4406	W5DZ64	Acyl carrier protein	17	2	14.6	5.94
55	1.4369	W5H631	60S ribosomal protein L27	39	2	15.5	10.48
56	1.4254	I7KM78	Gamma-gliadin OS=Triticum aestivum GN=GID-HE1 PE=2 SV=1	8	2	38	7.24
57	1.4076	W5EKI0	beta-amylase	35	4	61.1	5.15
58	1.4054	A0A1D5Z4H2	Prefoldin subunit 4	39	3	14.4	4.46
59	1.3913	A0A1D5YSI2	Transmembrane 9 superfamily member	10	2	67.6	8.65
60	1.3823	A0A1D5VHR2	Reticulon-like protein	20	4	35	7.3
61	1.3751	A0A125RLL8	MBD6-5AS	52	8	45	4.49
62	1.3670	D3JUT3	xylanase inhibitor protein I	29	8	33.3	8.47
63	1.3665	Q1XIR9	4-hydroxy-7-methoxy-3-oxo-3,4-dihydro-2H-1,4-benzoxazin-2-yl glucoside beta-D-glucosidase 1a, chloroplastic	60	9	64.5	7.02
64	1.3634	A0MAU4	Lipid transfer protein	25	3	18.8	8.12
65	1.3366	C1K737	multiprotein bridging factor 1	32	4	15.7	9.88
66	1.3365	Q5I5K7	small GTP-binding protein	36	2	22.9	7.36
67	1.3350	A0A1D5S627	peroxidase	7	2	41.9	7.64
68	1.3086	A0A1D5VA69	NADH-cytochrome b5 reductase	47	8	31	8.38
69	1.2954	A0A1D6CA75	Acyl-coenzyme A oxidase	9	5	75.6	8.06
70	1.2915	Q06I91	fasciclin-like protein FLA15	18	4	29.4	8.95
71	1.2912	A0A1D5SEA5	Eukaryotic translation initiation factor 3 subunit D	22	7	65.7	5.72
72	1.2890	W5A7D8	carboxypeptidase	19	4	53.2	5.25
73	1.2613	A0A1D5UM70	isocitrate dehydrogenase	9	2	53.5	8.27
74	1.2564	F2VQK3	Atp4-1	15	3	21.6	9.44
75	1.2532	Q0KIW2	glycine-rich RNA-binding protein	52	2	16	6.76
76	1.2488	A0A1D6BD14	glyceraldehyde-3-phosphate dehydrogenase	34	2	43.7	8.27
77	1.2122	A0A077S025	Protein ROOT HAIR DEFECTIVE 3 homolog	14	7	90.2	5.54
78	1.1520	C7AE91	Blue copper protein	24	4	17.4	5.31
79	1.1514	A0A1D6BGW9	Delta-aminolevulinic acid dehydratase	40	6	49.5	7.83
80	1.1415	A0A1D5YN81	Polyadenylate-binding protein	27	9	70.7	7.37
81	1.1394	Q7XY22	Mitochondrial pyruvate carrier	42	4	12.3	9.5
82	1.1302	A0A1D6BLG4	aspartate aminotransferase	23	2	63	8.02
83	1.1101	W5FPB7	pectinesterase	24	3	60.7	7.62
84	1.1098	A0A1D5RSC7	Alpha-galactosidase	25	7	44.7	7.96
85	1.1086	A0A1D5WFY2	small ubiquitin-related modifier	42	3	11.6	5.55
86	1.1008	Q7XYE0	NADPH-dependent mannose 6-phosphate reductase	29	2	30.1	6.9
87	1.0878	Q75QN8	cold shock domain protein 3	55	3	21.5	6.1
88	1.0876	A0A1D5SY22	pyruvate kinase	15	4	76.1	6.09
89	1.0765	C8CK09	phosphomannomutase	11	2	28.4	6.04
90	1.0763	A0A1D6CV93	peroxidase	22	4	38.4	4.86
91	1.0722	A0A1D5TCZ2	pectin acetylesterase	16	5	42.6	7.66
92	1.0634	W5CWG9	Sulfotransferase	18	5	37.8	6.74
93	1.0621	A0A1D5SX23	FRIGIDA-like protein	9	3	68	8.28
94	1.0511	W4ZQS8	Mitogen-activated protein kinase	20	5	42.8	6.16
95	1.0460	A0A1D5SAE3	60S ribosomal protein L18a	37	2	21.2	10.35

96	1.0336	A0A1D5Y470	Adenylyl-sulfate kinase	17	3	31	8.78
97	1.0255	A0A1D5UXT7	cysteine proteinase inhibitor	54	5	13.1	10.13
98	1.0218	Q6V959	ribosomal protein L3	33	7	44.6	10.07
99	1.0103	Q5EWZ1	Actin	62	2	41.7	5.4
100	1.0071	B2BA42	V-type proton ATPase subunit	28	6	40.7	5
101	1.0000	A0A1D6AZU5	peroxidase	62	3	34.5	7.83
102	0.9723	A0A1D5U388	proliferating cell nuclear antigen	45	8	30.8	4.84
103	0.9536	A0A1D6BNE8	Proteasome subunit beta type	24	2	22.9	5.44
104	0.9531	A0A1D5YUF6	Probable bifunctional methylthioribulose-1-phosphate dehydratase/enolase-phosphatase E1	13	2	56.7	6.44
105	0.9488	P69415	Photosystem I iron-sulfur center	64	5	8.9	6.92
106	0.9470	A0A1D6C0X8	ribonucleoside-diphosphate reductase	12	6	92.9	7.77
107	0.9462	W5GP51	peroxidase	52	4	34.8	8.18
108	0.9399	A0A1D5WQ86	peroxidase	37	3	35.4	8.44
109	0.9274	A0A1D5SSP1	Chlorophyll a-b binding protein, chloroplastic	94	2	16.9	4.88
110	0.9159	A0A1D6DKV5	Inositol-tetrakisphosphate 1-kinase	11	2	36.7	5.16
111	0.9126	A0A1D6BSN8	plastocyanin	46	2	15.6	5.87
112	0.9083	Q944C6	GTP-binding nuclear protein	36	6	25.1	7.12
113	0.9055	Q8W431	Sucrose:fructan 6-fructosyltransferase	37	3	68.4	5.5
114	0.9050	A0A1D5SHA7	D-3-phosphoglycerate dehydrogenase	25	8	64.7	8.44
115	0.8941	A0A1D6CMR8	peptidyl-prolyl cis-trans isomerase	28	5	23.4	9.57
116	0.8898	W5FIG3	ribulose-phosphate 3-epimerase	40	3	24.4	5.82
117	0.8632	Q8GVD3	thioredoxin	42	4	12.7	5.49
118	0.8519	A0A1D5YYQ8	Dirigent protein	62	3	12.3	6.7
119	0.8470	M4VSR0	low temperature-responsive RNA-binding protein	64	3	15.9	5.6
120	0.8425	W5F0H0	aminopeptidase	6	2	85.7	8.19
121	0.8347	W5D6S5	S-formylglutathione hydrolase	37	7	31.6	6.61
122	0.8325	A0A1D6CY42	peptidylprolyl isomerase	24	10	61.9	5.4
123	0.8197	A0A1D5V2T1	Cellulose synthase	12	7	123.1	7.24
124	0.8086	A0A172WCB1	cold-responsive LEA/RAB-related COR protein	24	2	17.2	4.96
125	0.8079	W5EQI5	Dirigent protein	14	2	18.8	8.09
126	0.8028	A0A1D5TM13	Pyruvate dehydrogenase E1 component subunit alpha	36	11	45.9	6.84
127	0.8011	W5DZ58	Coatomer subunit gamma	20	5	99.6	5.2
128	0.7962	A0A1D5TC74	peroxidase	23	4	34.2	4.84
129	0.7832	A0A1D5UUN4	small ubiquitin-related modifier	48	2	11.1	5.27
130	0.7679	A0A1D6DEQ5	peroxidase	65	2	34.8	8.18
131	0.7601	A0A1D5RTN9	proline iminopeptidase	10	2	44.4	7.85
132	0.7581	Q9FXQ9	TaWIN1	52	3	29.4	4.83
133	0.7566	A0A1D6D1F6	sucrose synthase	13	3	92.6	6.27
134	0.7550	A0A1D5SRA5	Coatomer subunit gamma	33	16	98.7	5.14
135	0.7422	A0A1D6CVC4	Ferredoxin	64	4	15.3	4.68
136	0.7412	A0A1D5SW54	ATP-dependent Clp protease proteolytic subunit	48	6	31.8	5.4
137	0.7407	A0A1D5VCF7	beta-glucosidase	6	2	54.8	5.58
138	0.7351	A0A1D5UAF0	Chlorophyll a-b binding protein, chloroplastic	64	2	17.1	5.5
139	0.7350	A0A1D6CMU2	Beta-galactosidase	22	3	78.5	8.1
140	0.7338	A0A1D5UX72	glycosyltransferase	12	2	53.6	6.09
141	0.7131	A0A1D5RZ51	beta-hexosaminidase	21	2	61.9	6.62
142	0.7088	C6K7G3	Lipoxygenase	48	2	96.5	6.65
143	0.7078	W5F8V5	Eukaryotic translation initiation factor 3 subunit E	36	9	50.7	5.69
144	0.6963	A0A1D6AFC1	peroxidase	44	8	33.5	8.32

145	0.6796	A0A1D5XH74	Beta-galactosidase	22	5	92.4	6.68
146	0.6794	A0A1D6D5D4	plastocyanin	46	2	15.6	5.87
147	0.6730	A0A1D5XGW1	Transmembrane 9 superfamily member	9	2	67.6	7.25
148	0.6625	M4PPG8	glycerol kinase	11	4	57.2	5.63
149	0.6611	A0A077KS83	aquaporin	18	4	25.8	6.52
150	0.6603	Q41518	single-stranded nucleic acid binding protein	72	4	16.2	5.25
151	0.6579	A0A1D5VSY7	peroxidase	14	3	37.5	8.47
152	0.6578	W5D5R6	cold induced protein	44	4	16.2	4.98
153	0.6560	A0A1D5XC94	Histone H2A	46	2	17.9	10.78
154	0.6516	A0A1D5SY80	60S ribosomal protein L36	15	2	14.6	11.11
155	0.6446	W5F8S6	Succinate dehydrogenase	26	10	68.2	6.57
156	0.6427	A0A075VVI2	50S ribosomal protein L22, chloroplastic	18	2	18.2	10.93
157	0.6411	A0A1D6SBV0	T-complex protein 1 subunit gamma	31	12	60.9	6.33
158	0.6384	A0A1D6BGI5	Protein arginine N-methyltransferase	6	2	73.6	5.39
159	0.6220	A0A1D5Z7E3	Serine/threonine-protein phosphatase	13	2	35.2	5.33
160	0.6180	Q5TMB2	Xylanase inhibitor TAXI-IV	22	3	42	8.32
161	0.6142	A0A1D5ST37	malate dehydrogenase	57	13	36.5	6.1
162	0.6110	A0A1D6B9G2	Phenylalanine ammonia-lyase	57	23	80.4	6.49
163	0.6078	O21432	Ribosomal protein S2	6	2	40.9	9.36
164	0.6015	L7PE91	Farnesyl pyrophosphate synthase	22	4	40.5	5.06
165	0.6006	Q3S861	Pyridoxine biosynthesis protein	24	4	33.3	6.87
166	0.5992	A0A1D5RU17	guanosine nucleotide diphosphate dissociation inhibitor	27	5	55.6	5.57
167	0.5984	W5I774	sucrose synthase	43	22	92.3	6.09
168	0.5874	Q9S8J6	Basic protein 3	100	2	2.1	7.99
169	0.5874	A0A1D6DEW4	Chlorophyll a-b binding protein, chloroplastic	78	2	27.4	5.25
1	-0.5850	A0A1D6RQH6	pyruvate kinase	23	6	59	7.25
2	-0.5935	W5AAU0	Chalcone-flavonone isomerase family protein	11	2	28.8	9.42
3	-0.5958	S4Z3I3	50S ribosomal protein L16, chloroplastic	16	2	16.6	11.49
4	-0.5984	F4Y590	heat shock protein 90	21	9	81.2	5.05
5	-0.6015	A0A075W3Y0	ATP synthase subunit beta, chloroplastic	87	36	53.8	5.22
6	-0.6035	W5FVU7	Proteasome subunit beta	47	8	27.5	7.23
7	-0.6056	A0A1D5SQI2	Nucleoside diphosphate kinase	16	4	35.8	9.31
8	-0.6061	A0A1D5UG33	Non-specific lipid-transfer protein	24	2	18.4	9.42
9	-0.6081	A0A1D5XGW0	deoxymugineic acid synthase	12	2	35.2	7.42
10	-0.6116	A0A1D6CEQ4	Probable alanine--tRNA ligase, chloroplastic	22	4	115.2	6.43
11	-0.6140	A0A1D5URM3	Glutamyl-tRNA(Gln) amidotransferase subunit B, chloroplastic/mitochondrial	29	6	60.6	6.43
12	-0.6159	A0A1D6CIV2	glycosyltransferase	5	2	49.5	5.6
13	-0.6184	A0A1D6ABL5	Histone H2A	44	2	14.4	10.67
14	-0.6184	A0A1D5YMU3	Pyruvate dehydrogenase E1 component subunit beta	28	2	40.2	5.38
15	-0.6191	P12073	DNA-directed RNA polymerase subunit alpha	22	2	38.8	7.06
16	-0.6193	A0A1D6BUR5	peptidylprolyl isomerase	28	4	24.8	7.55
17	-0.6202	A0A1D5ZAK3	ferritin	32	2	30.6	6.93
18	-0.6219	A0A1D6SCC4	3-oxoacyl-	41	2	48.4	7.4
19	-0.6249	A0A1D5UHC9	3-isopropylmalate dehydrogenase	38	8	41.9	6.11
20	-0.6315	A0A1D5YUD8	Calcium-transporting ATPase	5	4	111	6.09
21	-0.6384	A0A1D6S7C8	phosphoribulokinase	63	18	45.1	6.05
22	-0.6414	A0A1D5SR31	phosphotransferase	41	3	54.7	6.68
23	-0.6417	A0A1D6DKW7	Proteasome subunit alpha type	39	2	25.9	4.7
24	-0.6428	A0A1D5XDZ3	40S ribosomal protein SA	31	3	40.8	6.37

25	-0.6439	A0A0H4LXI0	catalase	36	8	56.4	7.25
26	-0.6448	Q9XPS8	DNA-directed RNA polymerase subunit beta'	4	2	78.2	8.95
27	-0.6480	A0A1D5ST23	ribosomal protein	29	10	37.5	8.16
28	-0.6566	A0A1D6BL90	Inositol polyphosphate multikinase	8	2	38.9	7.55
29	-0.6599	A0A1D5WDB9	Diphosphomevalonate decarboxylase	7	2	46.1	6.93
30	-0.6618	A0A1D6S0C4	Adenylosuccinate synthetase, chloroplastic	22	8	53	7.85
31	-0.6662	Q95H61	30S ribosomal protein S4, chloroplastic	34	8	23.3	10.98
32	-0.6676	P17933	30S ribosomal protein S2, chloroplastic	36	7	27	10.02
33	-0.6760	Q8RW00	glutathione transferase	34	6	26.4	5.1
34	-0.6799	W5BH99	GrpE protein homolog	43	7	31.3	6.01
35	-0.6882	A0A1D5WYS2	peroxidase	22	5	36.9	8.13
36	-0.6911	A0A1D6B0V1	Histone H2A	51	2	16.3	10.68
37	-0.6935	S4WGH0	Argonaute 4	6	3	105.5	8.97
38	-0.6995	Q95H43	NAD(P)H-quinone oxidoreductase subunit 1, chloroplastic	13	4	40.4	4.88
39	-0.6999	A0A1D5UUT2	Glucose-6-phosphate 1-dehydrogenase	8	2	65.8	8.21
40	-0.7109	S4Z098	ATP synthase subunit b, chloroplastic	37	5	21.4	9.32
41	-0.7134	Q95H51	50S ribosomal protein L14, chloroplastic	55	6	13.5	8.66
42	-0.7252	A0A1D6AUR0	Glucose-6-phosphate 1-dehydrogenase	15	5	64.8	8.24
43	-0.7290	A0A1D6AFZ1	Serine/threonine-protein phosphatase 2A 55 kDa regulatory subunit B	11	3	57.1	6.07
44	-0.7297	A0A1D5XSG4	aminopeptidase	24	2	98.9	5.24
45	-0.7318	W5GZC2	Transmembrane 9 superfamily member	4	2	74.2	6.79
46	-0.7325	Q5XUV3	Chloroplast inositol phosphatase-like protein	45	10	32.3	9.19
47	-0.7368	A0A1D6BQY9	tubulin beta chain	56	4	50.2	4.81
48	-0.7444	A0A1D6BMJ6	fructose-bisphosphate aldolase	60	3	37.9	6.8
49	-0.7453	A0A1D6AVF7	peptidylprolyl isomerase	17	3	24.7	9.6
50	-0.7574	A0A1D5UUZ0	peptidylprolyl isomerase	17	2	27.5	9.58
51	-0.7658	A0A1D5SR02	carboxypeptidase	14	4	48.1	7.18
52	-0.7697	D3K1B4	Ozone-responsive stress-related protein	33	2	8.7	9.25
53	-0.7710	A0A1D5SYC3	phosphotransferase	8	2	55.2	6.73
54	-0.7894	Q9XPT0	ATP synthase subunit a, chloroplastic	37	5	27.3	5.47
55	-0.7909	Q95H62	30S ribosomal protein S14, chloroplastic	22	2	12.2	11.37
56	-0.7964	A0A1D6DD56	alpha-amylase	7	4	97.4	5.76
57	-0.8058	W5E7H1	chorismate synthase	31	7	47.1	7.46
58	-0.8147	A0A1D6D1I0	Starch synthase, chloroplastic/amyloplastic	19	8	72	6.15
59	-0.8156	A0A075TNZ7	50S ribosomal protein L2, chloroplastic	40	8	30.5	11.37
60	-0.8183	M1PP16	Immunity resistance-related protein	15	3	31.2	5.05
61	-0.8254	A0A1D6AAQ1	Elongation factor Tu	67	23	50.4	6.28
62	-0.8254	C6YBD7	Chloroplast ribulose-1,5-bisphosphate carboxylase activase	65	3	40	6.93
63	-0.8273	A0A1D5YV20	sulfurtransferase	31	2	35.8	5.92
64	-0.8313	Q58QF6	delta-1-pyrroline-5-carboxylate synthase	18	9	77.7	6.6
65	-0.8403	A0A1D6D5W3	Probable alanine--tRNA ligase, chloroplastic	27	4	106.9	6.27
66	-0.8404	F4Y594	heat shock protein 90	49	3	80.4	5.06
67	-0.8494	D2KZ12	3-ketoacyl-CoA thiolase-like protein	45	2	47.9	7.96
68	-0.8503	A0A1D5S2P8	glucose-6-phosphate isomerase	48	3	62.3	7.43
69	-0.8524	A0A1D5UKJ1	Zeaxanthin epoxidase, chloroplastic	17	8	71.7	7.24
70	-0.8556	A0A1D5XKY8	coatomer subunit alpha	28	2	135.7	7.01
71	-0.8568	A0A077RQZ0	NAD(P)H-quinone oxidoreductase subunit I, chloroplastic	19	3	21	7.58
72	-0.8607	A0A1D5WPC9	glycine cleavage system P protein	76	3	99.5	6.43

73	-0.8613	A0A1D5XIE9	glycosyltransferase	6	2	54.6	6.43
74	-0.8725	A0A1D6ALH3	peroxidase	34	6	36.6	8.05
75	-0.8778	A0A1D5Z4G2	Vacuolar protein sorting-associated protein 35	16	8	89.8	5.34
76	-0.8792	Q95H42	NAD(P)H-quinone oxidoreductase subunit H, chloroplastic	52	15	45.7	5.67
77	-0.8813	A1YE31	Ribosomal protein L3-A3	30	4	44.6	9.95
78	-0.8822	S4Z1Z6	cytochrome b6	23	4	26	9.16
79	-0.8837	A0A1D6D7R5	peroxidase	24	2	42.8	7.18
80	-0.8896	G9HXG8	Isoamylase 3	9	4	86.2	6.54
81	-0.8929	A0A1B1QEA4	CCD-D1	17	8	61.7	6.28
82	-0.8980	A0A1D5V1G5	Chlorophyll a-b binding protein, chloroplastic	38	5	31.1	5.24
83	-0.8991	W5GDZ8	4-hydroxyphenylpyruvate dioxygenase	17	5	46.3	5.78
84	-0.9029	W5HZ68	40S ribosomal protein S27	44	3	9.6	8.72
85	-0.9063	A0A1D6AHV5	Terpene cyclase/mutase family member	8	4	86.1	6.29
86	-0.9092	A0A1D5YL67	glyceraldehyde-3-phosphate dehydrogenase	59	2	46.9	6.47
87	-0.9097	P24065	Photosystem II CP47 reaction center protein	42	14	56.1	6.54
88	-0.9181	A0A1D6CWU9	hexosyltransferase	6	2	58.8	9.45
89	-0.9295	D5MTE1	glycosyltransferase	35	4	49.4	5.6
90	-0.9444	Q8RW01	glutathione transferase	12	2	25.2	5.34
91	-0.9446	A0A1D6B9P2	NAD(P)H-hydrate epimerase	15	5	64.6	8.29
92	-0.9448	P69443	ATP synthase epsilon chain, chloroplastic	77	5	15.2	5.29
93	-0.9462	A0A1D5W5F3	Uricase	7	2	38.3	8.38
94	-0.9543	Q9XPS7	DNA-directed RNA polymerase subunit beta	10	7	121.6	8.81
95	-0.9545	B2CHJ7	Photosystem II D2 protein	38	9	39.5	5.55
96	-0.9609	I3NM60	putative 30S ribosomal protein S13	26	5	19.1	10.45
97	-0.9814	A0A1D6BAR0	carboxypeptidase	6	2	52.8	7.56
98	-0.9881	A0A1D5WVE3	Non-specific lipid-transfer protein	21	2	20.2	9.19
99	-0.9908	Q75RZ2	Putative caffeoyl CoA O-methyltransferase	13	3	29.1	5.21
100	-0.9920	A7J2I3	Plasma membrane intrinsic protein	29	2	29.9	9.17
101	-0.9958	A0A1D6BWT2	Rhomboid-like protein	9	2	35.7	8.66
102	-1.0008	A0A1D5WG52	H/ACA ribonucleoprotein complex subunit	13	2	23.4	11.47
103	-1.0018	A0A1D5X268	ferredoxin--NADP reductase	52	5	39.9	8.25
104	-1.0041	A0A1D5YVM4	Nucleoside diphosphate kinase	30	4	24	9.32
105	-1.0091	A0A1D5VHM9	ATP-dependent Clp protease proteolytic subunit	14	3	34.7	9.14
106	-1.0118	A0A1D5UTK1	GrpE protein homolog	25	4	37.2	7.4
107	-1.0189	A0A1D5X2X1	Lipoxygenase	16	8	99.4	6.61
108	-1.0229	D8L9S2	glutamate decarboxylase	44	3	54.1	5.78
109	-1.0298	A0A1D5YTJ8	Lipoxygenase	46	3	105.4	6.11
110	-1.0302	B0LXM0	S-adenosylmethionine synthase	37	2	43.2	5.88
111	-1.0377	A0A096URP1	Chlorophyll a-b binding protein, chloroplastic	63	10	30.8	6
112	-1.0379	Q35980	30 kDa subunit of complex I NADH dehydrogenase	12	3	33.6	9.5
113	-1.0583	W5AGK9	Nucleoside diphosphate kinase	70	2	16.7	6.8
114	-1.0591	Q84N29	Probable non-specific lipid-transfer protein 3	36	2	12.3	9.42
115	-1.0648	Q0Z9W3	histone deacetylase HDAC2	22	4	33.4	4.82
116	-1.0718	W5BBF4	Lipoxygenase	41	10	101.8	6.35
117	-1.0730	A0A1D5YP74	Mg-protoporphyrin IX chelatase	27	15	82.6	5.58
118	-1.0745	Q6EZE8	sucrose-phosphate synthase	17	2	115.1	6.2
119	-1.0762	A0A1D5XE21	phosphoserine aminotransferase	27	3	42.4	8.85
120	-1.0811	Q9ATG4	Non-specific lipid-transfer protein	37	3	11.3	9.2
121	-1.0820	A0A1D6AWF0	Chlorophyll a-b binding protein, chloroplastic	56	9	29.3	8.75

122	-1.0946	A0A1D5YPG2	malate dehydrogenase	60	9	37.2	8.18
123	-1.0981	A0A1D5SUC2	ATP-dependent Clp protease proteolytic subunit	10	2	42.9	9
124	-1.1013	Q95H55	30S ribosomal protein S18, chloroplastic	14	2	20.5	11.99
125	-1.1162	A0A0C4BJ37	Chlorophyll a-b binding protein, chloroplastic	37	3	31.1	5.35
126	-1.1267	A0A1Z1D7T0	NADH-plastoquinone oxidoreductase subunit 5	4	3	82.7	8.7
127	-1.1368	A0A1D6CMN4	glutathione synthetase	8	3	62.8	6.27
128	-1.1371	D3K4D8	ATP synthase subunit	57	3	39.7	8.02
129	-1.1484	W5CQ97	cysteine proteinase inhibitor	39	2	26.7	6.87
130	-1.1886	F8WTQ7	Delta-aminolevulinic acid dehydratase	59	3	46.2	5.83
131	-1.1942	A0A1D5UP29	Lipoxygenase	33	5	101.8	6.43
132	-1.1954	A0A1D5VEA1	amidophosphoribosyltransferase	9	4	59.1	6.52
133	-1.2273	E6YBC7	Plasma membrane intrinsic protein	43	6	30.7	8.81
134	-1.2497	L0N608	cell wall invertase	20	7	64.5	8.28
135	-1.2566	A0A1D6S8Q3	Lipoxygenase	27	13	102.3	6.14
136	-1.2580	A0A1D6AB44	Lysine--tRNA ligase	9	4	65.5	6.51
137	-1.2589	A0A1D6BSD2	Chlorophyll a-b binding protein, chloroplastic	72	2	28.4	5.27
138	-1.2847	Q00810	Cytochrome c oxidase subunit 2	12	2	29.5	4.93
139	-1.2858	Q9M4V3	ribulose bisphosphate carboxylase activase B	63	2	47.8	7.36
140	-1.3002	P69555	Photosystem II reaction center protein H	23	2	7.8	8.57
141	-1.3019	A0A1D5T5A4	40S ribosomal protein S7	35	2	22.2	9.82
142	-1.3163	O04437	glutathione S-transferase	15	2	23.7	6.52
143	-1.3204	A0A1D5RSS8	Tyrosine--tRNA ligase	16	2	53.4	7.88
144	-1.3328	C0KTA6	fructose-bisphosphate aldolase	76	2	42	6.16
145	-1.3363	W4ZR59	mitochondrial fission 1 protein	17	2	18	7.25
146	-1.3382	A0A1D5UA20	Lon protease homolog, mitochondrial	9	5	107.4	5.58
147	-1.3614	W5DZR3	ATP-dependent Clp protease proteolytic subunit	20	4	30.2	8.59
148	-1.3984	W5FL28	Lipoxygenase	42	6	105.4	6.11
149	-1.4136	A0A1D5YU28	Elongation factor Ts, mitochondrial	45	6	91	4.87
150	-1.4300	A0A1D5X3S5	ferritin	22	3	28.3	5.99
151	-1.4507	W5B421	lactoylglutathione lyase	15	2	25.8	8.65
152	-1.4604	A0A1D6BU87	carboxypeptidase	12	3	50.9	7.96
153	-1.4646	A0A077S0C9	Peptide deformylase	15	2	27.5	8.57
154	-1.4831	A0A1D5TDS2	acetyl-coenzyme A synthetase	7	2	80.8	6.54
155	-1.5161	A4ZCD1	GTP-binding protein	21	2	58.9	5.72
156	-1.5514	A0A077KVU8	Tonoplast intrinsic protein	16	2	25	5.71
157	-1.5578	Q5CAL1	putative glucan endo-1,3-beta-D-glucosidase	54	2	34.2	7.33
158	-1.5912	N0E6R8	DEAD-box ATP-dependent RNA helicase, putative	8	3	90.6	10.11
159	-1.6208	A0A1D6BFL6	glycosyltransferase	16	2	51.6	5.82
160	-1.6322	P69386	Cytochrome b559 subunit alpha	37	4	9.4	4.78
161	-1.6559	W4ZUA0	NADH-cytochrome b5 reductase	12	2	31	8.12
162	-1.6725	A0A1D5U2X7	Glutathione peroxidase	15	2	19.3	7.84
163	-1.6765	Q4Z8L8	class II chitinase	39	2	28.2	8.37
164	-1.6844	W5ASP5	Lipoxygenase	32	6	101.4	6.28
165	-1.6926	F8S6U7	Pathogenesis-related protein 1	36	3	18.9	7.72
166	-1.7330	W5G4K3	Lipoxygenase	46	3	105.3	6.1
167	-1.7341	C1IP75	lycopene epsilon cyclase 3B	4	2	59.6	7.31
168	-1.8105	W5FWD9	peroxidase	11	2	39	8.75
169	-1.9432	W5G3J1	Lipoxygenase	12	6	107.6	6.35
170	-1.9760	A0A1D6AYW9	Lipoxygenase	4	2	99.4	6.32
171	-2.1561	A0A1D5ZB96	glucose-1-phosphate adenylyltransferase	11	2	54.6	6.07

172	-2.1627	P29557	Eukaryotic translation initiation factor 4E-1	22	3	24	5.62
173	-2.2241	P58420	NAD(P)H-quinone oxidoreductase chain 4, chloroplastic	4	2	56.3	8.51
174	-2.2910	A0A1D6C1S7	Elongation factor Ts, mitochondrial	7	2	41.3	8.28
175	-2.4669	A0A1D6DBP8	beta-amylase	13	5	72.1	6.04
176	-2.7471	A0A1D5UP28	Small nuclear ribonucleoprotein-associated protein	13	3	28	11.3
177	-2.8298	W5B1R3	glycosyltransferase	13	3	54	5.26
178	-2.9376	A0A1D5T678	Clathrin light chain	11	2	33.2	4.86
179	-3.5203	A0A1D6A5H9	phosphate transporter	6	2	59.3	9.33
180	-3.9925	A0A1D5UPH3	Endoglucanase	10	3	60.3	9.39

Cov, coverage; MP, matched peptides; MW, molecular weight; and calc pI, calculated pI.

Table S4. A list of amino acids in wheat leaves treated with or without plant-derived smoke solution under flooding compared with control.

Peak No	Amino acids	Control average (mg/ 100 g)	Control S.D.	Food average (mg/ 100 g)	Flood S.D.	Flood + Smoke average (mg/ 100 g)	Flood + Smoke S.D.	Flood/ Control	Flood + smoke/ Control
1	P-Ser	0.366	0.056	0.377	0.069	0.544	0.064	1.031	1.488
2	Tau	0.099	0.058	0.129	0.036	0.098	0.040	1.299	0.983
3	PEA	0.627	0.171	0.173	0.116	0.212	0.144	0.275	0.337
5	Asp	1.844	0.543	0.601	0.254	1.259	1.031	0.326	0.683
6	Thr	1.491	0.336	2.497	0.108	2.627	0.143	1.674	1.761
7	Ser	4.353	0.722	0.720	0.222	1.171	0.608	0.165	0.269
8	Asn	34.831	14.364	40.511	14.476	45.247	7.783	1.163	1.299
9	Glu	5.767	1.468	ND		7.569	2.220	0.000	1.312
10	Gln	9.005	1.756	1.607	0.288	2.265	1.052	0.179	0.252
13	Gly	0.581	0.211	1.562	0.002	1.675	0.177	2.688	2.881
14	Ala	4.781	1.045	11.910	1.852	15.968	1.781	2.491	3.340
15	Cit	0.539	0.094	1.757	0.716	2.496	0.386	3.261	4.634
16	a-ABA	0.062	0.009	0.049	0.001	0.047	0.000	0.790	0.758
17	Val	1.663	0.503	4.282	0.518	5.327	0.522	2.575	3.203
18	Cys	ND		ND		ND		ND	ND
19	Met	0.336	0.045	0.645	0.150	0.649	0.093	1.921	1.935
21	Ile	0.857	0.265	2.718	0.250	3.281	0.337	3.173	3.830
22	Leu	0.706	0.154	4.121	0.339	4.468	0.655	5.840	6.332
23	Tyr	0.501	0.063	2.272	0.031	2.602	0.227	4.531	5.190
24	b-Ala	0.287	0.144	0.340	0.128	0.484	0.170	1.186	1.686
25	Phe	0.522	0.065	844.658	1191.010	3.294	0.404	1619.153	6.314
27	GABA	4.820	1.349	12.648	2.405	15.220	1.738	2.624	3.158
28	MEA	0.982	0.159	0.653	0.159	0.828	0.170	0.665	0.844
29	NH3	0.407	0.055	1.051	0.095	1.434	0.194	2.581	3.521
31	Orn	0.046	0.000	0.490	0.158	0.362	0.119	10.652	7.862
32	1M-His	0.450	0.067	1.057	0.215	1.291	0.275	2.347	2.867
33	His	1.078	0.331	2.234	0.223	2.552	0.338	2.073	2.368
34	Lys	0.875	0.235	4.186	0.362	4.674	0.512	4.785	5.343
36	Trp	0.381	0.072	0.699	0.012	0.856	0.119	1.836	2.246
39	Arg	0.466	0.056	1.257	0.651	0.914	0.048	2.700	1.963
